# Anlotinib Alleviates Renal Fibrosis via Inhibition of the ERK and AKT Signaling Pathways

**DOI:** 10.1155/2023/1686804

**Published:** 2023-02-18

**Authors:** Donglin Sun, Jing Guo, Weifei Liang, Yangxiao Chen, Xiangqiu Chen, Li Wang

**Affiliations:** ^1^Guangzhou Medical University, Guangzhou 510000, China; ^2^Qingyuan People's Hospital, The Sixth Affiliated Hospital of Guangzhou Medical University, Qingyuan 511500, China; ^3^Department of Urology, Shenzhen Hospital, Southern Medical University, Shenzhen 518100, China; ^4^Southern Medical University Affiliated Longhua People's Hospital, Nephrology Department, Shenzhen, 518020 Guangdong, China

## Abstract

**Purpose:**

We examined whether anlotinib can attenuate folic acid-induced and unilateral ureteral obstruction-induced renal fibrosis and explored the underlying antifibrotic mechanism.

**Materials and Methods:**

We have evaluated the effects of anlotinib on folic acid-induced and unilateral ureteral obstruction-induced renal fibrosis in mice through in vivo experiments of unilateral ureteral obstruction or folic acid-induced interstitial fibrosis and in vitro models of transforming growth factor-*β*1 induced HK-2 human renal proximal tubule cells. Serum renal function parameters and inflammatory cytokine levels were measured, and histological changes of renal injury and fibrosis were analyzed by HE staining and immunohistochemistry. Immunohistochemistry and Western blotting were used to determine the mechanism of action of anlotinib in ameliorating renal fibrosis.

**Results:**

Anlotinib improved proteinuria and reduced renal impairment in folic acid-induced mouse models of renal fibrosis. Anlotinib reduced tubular injury, deposition of tubular extracellular matrix, and expression of alpha-smooth muscle actin, transforming growth factor-*β*1, and cytosolic inflammatory factors compared with controls.

**Conclusions:**

Anlotinib ameliorated renal function, improved extracellular matrix deposition, reduced protein levels of epithelial-mesenchymal transition markers, and decreased cellular inflammatory factors. Anlotinib reduced renal injury and fibrosis by inhibiting the transforming growth factor-*β*1 signaling pathway through AKT and ERK channels.

## 1. Introduction

Chronic kidney disease (CKD), a global public health concern, often ends in serious conditions like renal failure and cardiovascular disease, posing a serious risk to human health and leading to premature death in 82% of patients [1]. As of today, the only options available to patients with end-stage renal disease are kidney transplantation and dialysis, imposing a huge economic burden on patients and society alike [2]. Therefore, it is crucial to find effective treatments to prolong the life span of CKD patients and reduce corresponding medical costs.

Progressive tubulointerstitial fibrosis is a common pathological change in chronic kidney disease [3] that eventually leads to renal failure. As the main component (about 90%) of the kidney is tubulointerstitium, the degree of tubulointerstitial fibrosis is a keen indicator of CKD prognosis. Given this, effective prevention or handling of tubulointerstitial fibrosis will contribute to improving the prognosis of CKD. Nevertheless, there are currently no effective drugs for the treatment of tubulointerstitial fibrosis. The identification of new therapeutic agents for patients with chronic kidney disease is therefore of utmost importance.

Renal tubular interstitial fibrosis is characterized by inflammation, extracellular matrix (ECM) deposition, loss of tubular cells, accumulation of fibroblast activation, and reduction of peritubular microvasculature [4, 5]. Pathological deposition of ECM, primarily collagen I and collagen IV, is a hallmark of renal fibrosis [6]. Epithelial-mesenchymal transition (EMT) is a major cause of renal interstitial fibrosis [7, 8] and manifests as the acquisition of a mesenchymal phenotype and myofibroblast function by renal tubular epithelial cells [9]. EMT reduces the expression of cell adhesion proteins like E-cadherin by renal epithelial cells while inducing fibroblasts to express wave proteins and alpha-smooth muscle actin (*α*-SMA) [7]. Renal fibrosis essentially evolves from inflammatory cell infiltration, and activated inflammatory cells not only produce several proinflammatory cytokines and chemokines such as chemokine ligand 2 (CCL-2)/monocyte chemotactic protein-1 (MCP-1) but also produce profibrotic cytokines like TGF-*β*1, which are regarded as major mediators in renal fibrosis pathogenesis [10]. Therefore, inhibiting the inflammatory response and reducing the fibrotic response and ECM deposition would be potential targets for the treatment of renal fibrosis [4].

Anlotinib hydrochloride (AL3818) is a novel multitargeted tyrosine kinase inhibitor (TKI) that has been reported to be effective in the treatment of cancers like non-small-cell lung cancer, osteosarcomas, and endometriomas [11, 12]. More importantly, it has been found to ameliorate the symptoms of mouse lung fibrosis by interfering with the TGF-*β* signaling pathway [13]. AL3818's target receptors include vascular endothelial growth factor receptors (VEGFR) 1 to 3, epidermal growth factor receptor (EGFR), fibroblast growth factor receptors (FGFR) 1 to 4, platelet-derived growth factor receptors (PDGFR) *α* and *β*, and stem cell factor receptor [14, 15]. TGF-*β*1 and fibroblast activation is essential in the pathogenesis of renal fibrosis, so we speculated that anlotinib could improve and prevent renal fibrosis, and no studies have been conducted on the effects of anlotinib on renal fibrosis and the associated mechanism of action.

Our study examined anlotinib's antifibrotic effects on fibroblast transdifferentiation induced by TGF-1, folic acid (FA), and unilateral ureteral obstruction (UUO) in healthy subjects. Anlotinib therapy was found to assist in reducing both in vitro and in vivo markers of fibrosis, and its antifibrotic effect is mediated by inhibition of AKT, ERK signaling channels, and TGF-*β*1 transduction. This study lays the groundwork for further exploring the therapeutic potential of anlotinib intervention in renal fibrosis.

## 2. Materials and Methods

### 2.1. Animals

Male C57BL/6 mice of 8 weeks were available at Rise Mice Biotechnology Co., Ltd. (Zhaoqing, China). Body weight was approximately 20-25 g. The mice were free to walk and drink under stable conditions of 25°C and 12 hours of light/dark. Experimental animals were cared for and used by the Guide for the Care and Use of Laboratory Animals, which has been approved by Southern Medical University Affiliated Longhua People's Hospital's Institutional Biomedical Research Ethics Committee.

### 2.2. FA Mouse Models

The FA nephropathy mouse models were established and randomly divided into 4 groups. In the control group, DMSO was injected intraperitoneally; in the control+anlotinib group, DMSO+1 mg/kg anlotinib was injected intraperitoneally; in the FA group, 25 *μ*g/g folic acid was injected intraperitoneally; in the anlotinib treatment group, 25 *μ*g/g folic acid and 1 mg/kg anlotinib were injected intraperitoneally. DMSO or anlotinib was administered consistently daily for 34 days, with FA administered on day 1 only. After 34 days, 24 h urine was collected and the mice were euthanized to obtain serum.

### 2.3. UUO Mouse Models

Prior to surgery, mice were anesthetized with 1.25% 2,2,2-tribromoethanol through intraperitoneal injection. Then, UUO was established by double ligation of the left ureter with 4-0 silk after the abdominal incision. The ureter of the sham-operated mice was exposed without ligation. Anlotinib was injected intraperitoneally at 1 mg/kg in the anlotinib-treated group daily for 7 days. After that, mice were executed, and urine, serum, and kidney tissues were collected for further analysis.

### 2.4. Cell Culture

ATCC (Manassas, VA) provided HK-2 human renal proximal tubule cells. An HK2 cell line was grown in a keratinocyte serum-free medium (Invitrogen) and tested at generations 10-13. Following pretreatment with/without anlotinib (2 *μ*M) for 4 h, cells were incubated with TGF-*β*1 for 48 h. Thereafter, cell lysates and supernatants were prepared as described below.

### 2.5. CCK8 Assay

The Cell Counting Kit 8 (Beyotime) was used in accordance with provided protocols. Briefly, 2000 cells were added into 96-well plates per well. 10 *μ*L CCK8 solution was added per cell. The cells were then cultured for 2 h at 37°C while protected from light. Data were collected by reading the optical density (OD) at 450 nm at Thermo Scientific Microplate Reader.

### 2.6. RNA Extraction and Real-Time RT-PCR

Kidney tissue was homogenized using TRIzol reagent (Life Technologies, 15596-026), and total RNA was extracted as directed by the manufacturer. The RNA was then converted to cDNA according to the instructions of the PrimeScript™ RT reagent Kit with gDNA Eraser (TaKaRa, RR047A). Real-time RT-PCR was performed by the Q7 RT-PCR detection system (Life Technologies) using SYBR® premix Ex Taq (TaKaRa, RR420A). Relative quantification (RQ) was derived from the cycling threshold (Ct) using the equation RQ = 2^−ΔΔCt^. The results were normalized to the expression of *β*-actin mRNA. The primers used are listed in Additional file 1.

### 2.7. Western Blotting

Kidney tissues were washed with precooled PBS, homogenized by a homogenizer, and then lysed in RIPA (Beyotime, P0013F), protease inhibitor mixture (Roche Diagnostics, 4693116001), phosphatase inhibitor mixture (Roche Diagnostics, 4906845001), and PMSF (Beyotime, ST506), then incubated on ice for 30 min, and the supernatant was collected by centrifugation at 13,000g for 15 min and stored at -80°C. Total protein concentration was estimated using the BCA Protein Assay Kit (Thermo Fisher Scientific, 23225). Protein samples (10-20 *μ*g) were diluted with 5x loading buffer, bathed in metal for 10-15 min, separated by 12.5% SDS polyacrylamide gel electrophoresis (SDS-PAGE), and then transferred to PVDF membranes (Millipore) for detection of specific antibody probes. The immunoreactive proteins were visualized by an enhanced chemiluminescence detection system (Millipore). The following primary antibodies were used: anti-collagen type I (anti-collagen I; rabbit; Abcam, ab260043), anti-collagen type IV (anti-collagen IV; rabbit; Abcam, ab6586), anti-TGF-*β*1 (rabbit; Abcam, ab215715), anti-ERK (rabbit; Abcam, ab184699), anti-p-ERK (rabbit; Abcam, ab201015), anti-AKT (rabbit; Abcam, ab8805), anti-p-AKT (rabbit; Abcam, ab38449), anti-*α* smooth muscle actin (anti-*α*-SMA; rabbit; Abcam, ab5694), and anti-GAPDH (KANGCHEN, KC-5G4). The secondary antibodies were used: peroxidase-conjugated goat anti-mouse IgG (H+L) (33201ES60, Yeasen) and peroxidase-conjugated rabbit anti-goat IgG (H+L) (33701ES60, Yeasen).

### 2.8. Histological and Immunohistochemical Analyses

The kidneys were fixed in 4% paraformaldehyde (Beyotime, P0099-500 mL) and embedded in paraffin. Paraffin-embedded mouse kidney sections (3 *μ*m thickness) were prepared following a routine procedure, where the sections were stained with hematoxylin and eosin (H&E) and Masson trichrome reagent (Service, G1006) separately. For immunohistochemical staining, the sections were deparaffinized and rehydrated, followed by antigen retrieval and blocking. Then, the tissue sections were incubated at 4°C overnight with diluted primary antibodies: anti-*α*-SMA (Abcam, ab5694), anti-F4/80 (Abcam, ab6640), and anti-CD163 (Abcam, ab182422). The secondary antibodies were applied, and a diaminobenzidine (DAB) solution was used as a chromogen. In addition, the sections were counterstained with hematoxylin (Sigma) to identify nuclei. The images were photographed at 200x and 400x by a general optical microscope (Carl Zeiss) and analyzed using Image-Pro Plus 6.0 software (Media Cybernetics, Inc.).

### 2.9. Statistical Analysis

Each experiment was repeated at least three times. Values are expressed as mean ± SD, and statistical analyses were performed using unpaired Student's *t*-test (two groups) and Kruskal-Wallis one-way analysis of variance (more than two groups). *P* values below 0.05 indicate a significant difference between groups.

## 3. Results

### 3.1. Anlotinib Ameliorates Renal Function in FA-Induced Mouse Models

**Figure 1:**
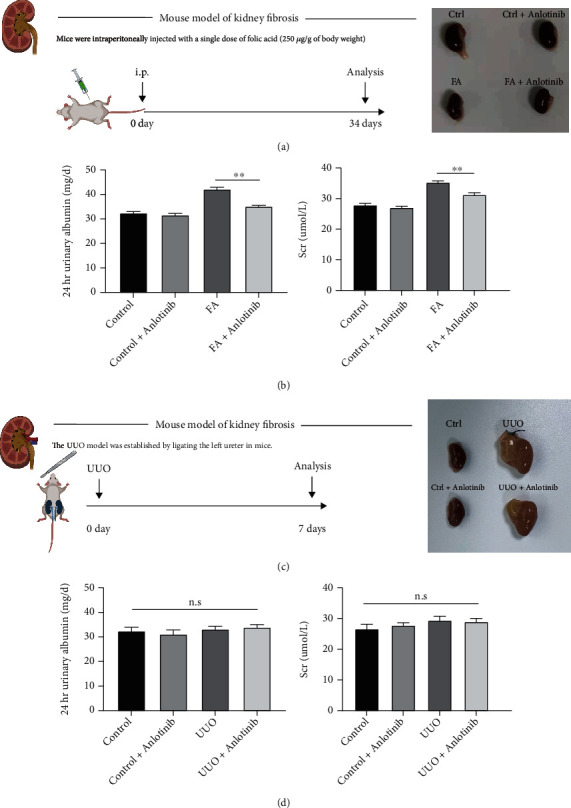
Anlotinib improved albuminuria and serum creatinine in an FA-induced mouse model. FA-induced mouse models of nephropathy were treated with or without anlotinib in this study. (a) Intervention dosing regimen of folic acid in an experimental mouse model of kidney fibrosis. (b) 24-hour urinary albumin excretion and serum creatinine were measured at 14 days after folic acid injection, and both were significantly increased and later reduced after the mice were treated with anlotinib in FA-induced mice compared to the control group. (c) Established UUO-induced mouse models of kidney fibrosis. (d) UUO-induced mouse models of nephropathy showed no significance when treated with or without anlotinib in 24-hour urinary albumin excretion and serum creatinine. Results are presented as mean ± SEM. ^∗∗^*P* < 0.01, n.s indicates not significant (*P* > 0.05), *n* = 3.

We examined the effect of anlotinib on renal function and fibrosis phenotype in vivo; we determined the dose to be 1 mg/kg for mice. Then, the cytotoxicity of anlotinib was evaluated by the CCK8 assay in vivo. Combined with cytotoxicity and mRNA levels of fibrosis-related indicators, the optimal concentration of anlotinib in vitro is 2 *μ*M (Supplementary Figure S1). In order to evaluate anlotinib's effects on urinary albumin, we established a control group and a FA group (Figure 1(a)) and measured 24-hour urinary albumin and serum creatinine levels at the time of execution. In the control group, as shown in Figure 1(b), there was no significant difference in urinary albumin and creatinine in mice with or without anlotinib; however, in the FA group, urinary albumin and creatinine were significantly reduced after anlotinib treatment (*P* value < 0.05). Meanwhile, we established UUO mouse models (Figure 1(c)) to investigate whether anlotinib could improve the renal function of mice. In the UUO group, anlotinib was found to exert no significant effect on the renal function of mouse models, as shown in Figure 1(d). Considering only the left ureter was ligated, the reason may be that the healthy contralateral kidney compensated and maintained the normal function of the whole kidney. According to these findings, anlotinib improves the recovery of renal function after toxic injury.

### 3.2. Anlotinib Inhibits Renal ECM Gene Expression and Reduces Tubulointerstitial Fibrosis

**Figure 2:**
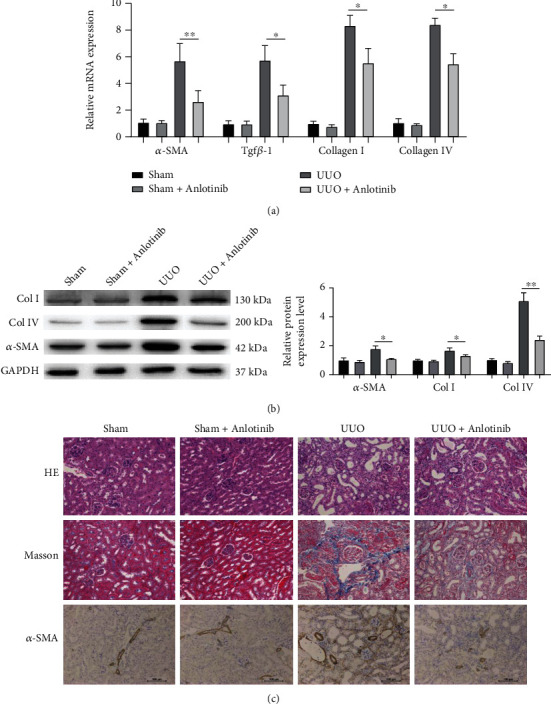
Anlotinib reduced the accumulation of extracellular matrix components and blocked EMT in UUO mice. (a) Quantitative RT-PCR was performed to determine the RNA expression of *α*-SMA, TGF-*β*1, collagen І, and collagen ІV in the kidney tissue of UUO mice. (b) Western blotting was performed to determine the protein expression of *α*-SMA, collagen І, and collagen ІV in the kidney tissue of UUO mice. (c) The representative photographs of the effects of anlotinib against OUU-induced pathological changes (HE and Masson stain) at day 14; interstitial fibrosis was evident in mouse kidney from the UUO group, compared with the sham group. The pathological changes were obviously relieved in the kidney of the anlotinib-treated group. This was immunohistochemical staining of *α*-SMA in the renal cortex from the experimental mice. (Bars, 100 *μ*m. All data are represented as mean ± SD. ^∗^*P* < 0.05 and ^∗∗^*P* < 0.01, *n* = 3. Scale bars, 100 *μ*m.).

Collagen (collagen I and collagen IV) is an essential part of the renal ECM, *α*-SMA is a key secretory protein of fibroblasts, and TGF-*β*1 is an underlying factor in renal fibrosis. In order to determine whether anlotinib regulates renal fibrosis, we examined the impact of anlotinib on type I and IV collagen expression, *α*-SMA, and TGF-*β*1. As shown in Figures 2(a) and 2(b), in the UUO mouse models, type I and type IV collagen and *α*-SMA and TGF-*β*1 expressions were observed in the kidneys of UUO-treated animals compared with the control group, and it was found to be significantly reduced in the anlotinib-treated group, hence its significant inhibition by anlotinib. In addition, we examined the effects of anlotinib on the expression of interstitial collagen fibers with HE and Masson staining and analyzed *α*-SMA with immunohistochemical staining and the expression of type I and IV collagen and *α*-SMA with Western blotting, whose results showed that the expression of type I and IV collagen and *α*-SMA protein in the anlotinib-treated group was significantly reduced compared with the UUO group (*P* < 0.05, Figure 2(b)). In addition, collagen deposition was significantly increased in the interstitial region of the kidney in UUO mice compared with the anlotinib-treated group (Figure 2(c)). Based on these data, anlotinib inhibits ECM production and inhibits renal fibrosis in the UUO mouse models.

### 3.3. Anlotinib Reduces Inflammatory Responses in Mouse Models of UUO

**Figure 3:**
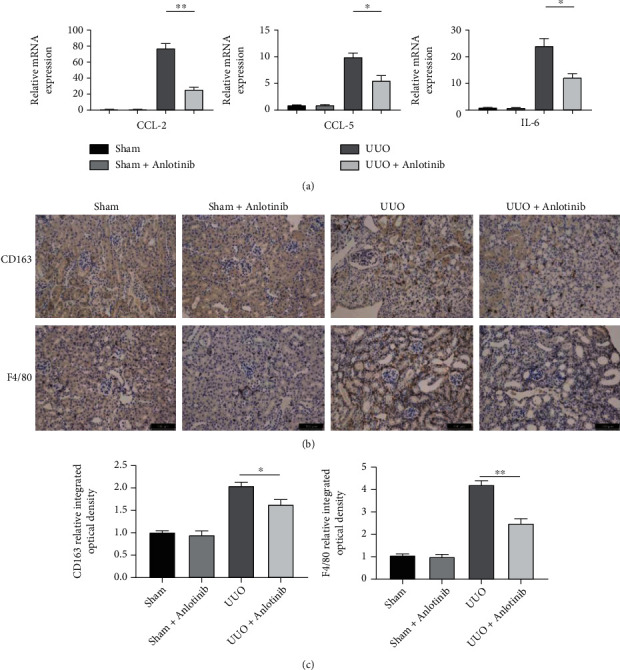
Anlotinib attenuated inflammatory responses in a UUO mouse model. (a) The mRNA expression of CCL2, CCL5, and IL-6 in the mouse kidneys was examined by real-time PCR. Quantitative RT-PCR showed increased mRNA expression of CCL2, CCL5, and IL-6 in UUO mice compared to the sham group. These markers of inflammation were reduced in the mice treated with anlotinib. (b, c) Immunochemical staining of CD163 and F4/80 in the kidney tissue of OUU mice. Results are presented as mean ± SEM. ^∗^*P* < 0.05 and ^∗∗^*P* < 0.01, *n* = 3.Scale bars: 100 *μ*m.

To investigate the effect of anlotinib on renal inflammation in UUO mice, we examined three proinflammatory cytokines CCL-2, CCL-5, and IL-6 in renal tissues along with two macrophage markers, F4/80 and CD163. As shown in Figure 3(a), CCL-2, CCL-5, and IL-6 expressions in the UUO group were significantly increased compared to the anlotinib-treated group, and mRNA expression of the three proinflammatory cytokines was attenuated by anlotinib. To further determine whether macrophage infiltration contributed to UUO-induced renal injury, F4/80 and CD163 expressions were assessed by immunohistochemical staining (Figure 3(b)), and anlotinib significantly inhibited the expression of F4/80 (*P* < 0.05) and CD163 (*P* < 0.01) (Figure 3(c)). These results indicate that anlotinib may reduce inflammation.

### 3.4. Anlotinib Suppresses TGF-*β*1-Stimulated Collagen and *α*-SMA Expression in Human Proximal Renal Tubular Cells

**Figure 4:**
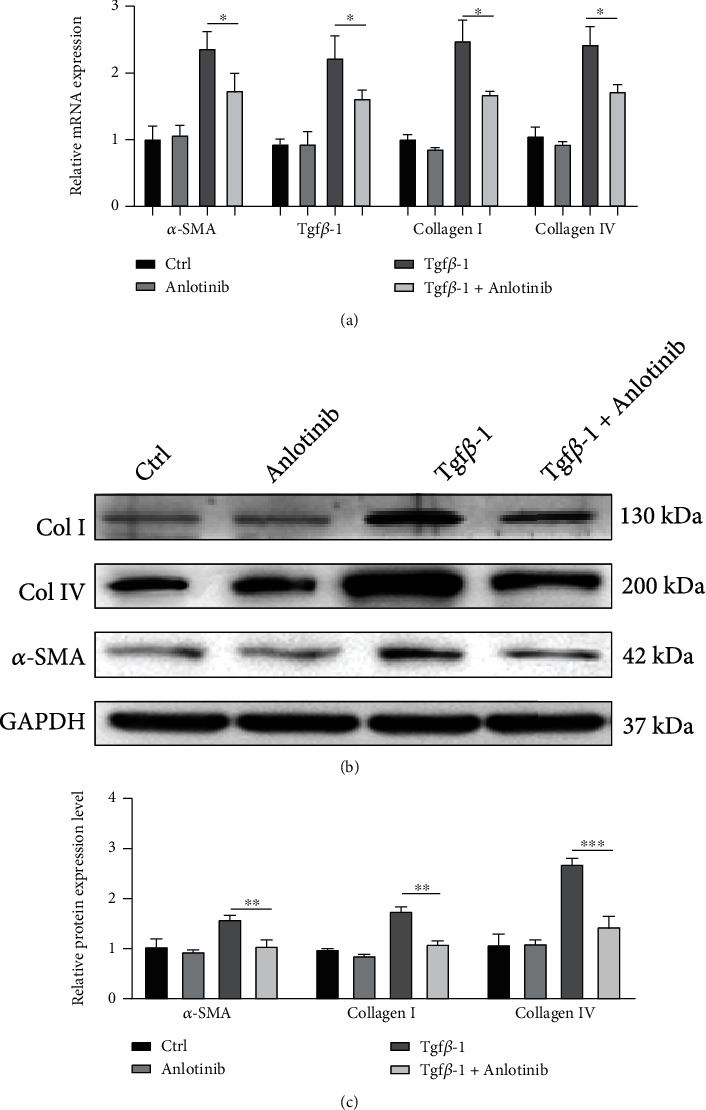
Anlotinib inhibited TGF-*β*1-stimulated collagen and *α*-SMA expression in human proximal tubular cells. (a) Human proximal tubular cells pretreated with/without anlotinib for 4 hours were incubated with TGF-*β*1 for 48 hours. Real-time RT-PCR results showed that anlotinib inhibited TGF-*β*1-induced *α*-SMA, collagen I, and collagen IV mRNA expression. (b, c) Western blot results showed that anlotinib inhibited the induction of *α*-SMA, collagen I, and collagen IV in TGF-*β*1-treated human proximal tubular cells. Results are presented as mean ± SEM. ^∗^*P* < 0.05, ^∗∗^*P* < 0.01 and ^∗∗∗^*P* < 0.001, *n* = 3.

TGF-*β*1, a key player in renal fibrosis [16], stimulates the upregulation of collagen and *α*-SMA expressions in renal tubular cells. To investigate the effect of anlotinib on ECM production after TGF-*β*1 exposure, we used real-time PCR and Western blotting to detect the expression of type I and type IV collagen and *α*-SMA. As shown in Figures 4(a)–4(c), TGF-*β*1 induced the mRNA expression of type I and type IV collagen and *α*-SMA in human proximal renal tubular cells, which decreased significantly in the presence of anlotinib (*P* < 0.05, Figure 4(a)). Further validation using Western blotting demonstrated that anlotinib reduced type I and type IV collagen and *α*-SMA expression in TGF-*β*1-exposed human proximal tubular cells (*P* < 0.01, Figures 4(b) and 4(c)).

### 3.5. Anlotinib Attenuates Renal Fibrosis by Inhibiting TGF-*β*1-Mediated ERK and AKT Pathways

**Figure 5:**
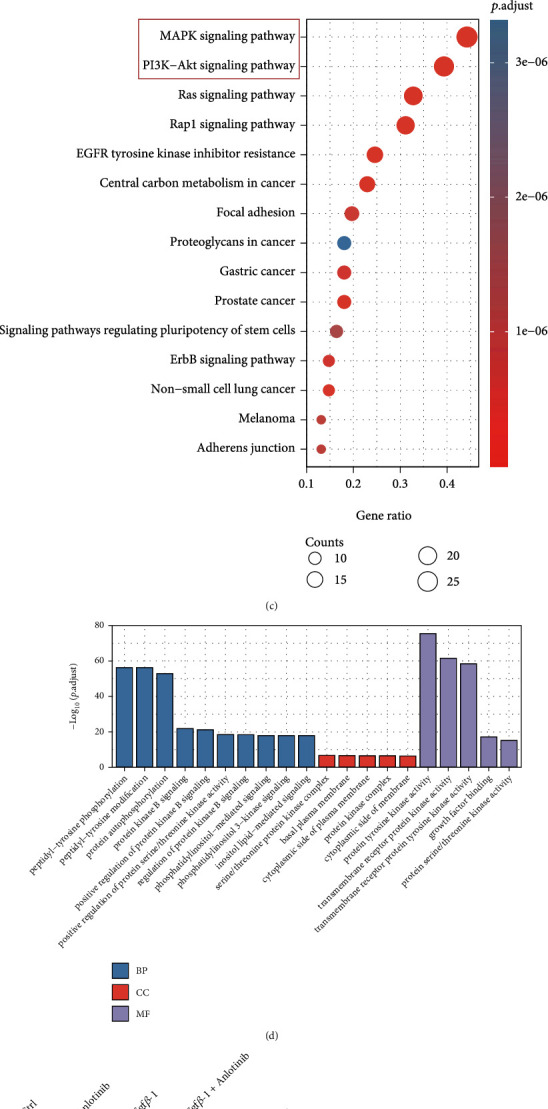
Anlotinib attenuated kidney fibrosis via the mediation of TGF-*β*1 signaling via ERK and AKT pathways. (a) Venn diagram showed the overlaps between anlotinib targets and renal fibrosis-related targets. (b) Protein-protein interaction (PPI) network of common targets between anlotinib and renal fibrosis. (c) The KEGG enrichment analysis of 70 targets of common targets. (d) The GO enrichment for each section is listed. (e, f) Western blot results showed that anlotinib inhibited TGF-*β*1-induced increases in p-AKT and p-ERK in human proximal tubular cells. Results are presented as mean ± SEM. ^∗^*P* < 0.05 and ^∗∗^*P* < 0.01, *n* = 3.

To explore anlotinib's potential mechanisms for treating renal fibrosis, we estimated the most probable molecular targets of anlotinib and obtained 100 potential targets by SwissTargetPrediction (Additional file 2) [17]. A total of 6549 targets (Additional file 3) associated with renal fibrosis were obtained from the GeneCards database. The intersection of targets was mapped by drawing Venn diagrams and constructing target networks (Figures 5(a) and 5(b)) for the purpose of elucidating the interactions between potential targets of anlotinib and renal fibrosis-related targets. Results indicated that the potential targets of anlotinib and renal fibrosis-related targets share a total of 70 common targets (Additional file 3) (Figure 5(a)). Protein interaction (PPI) analysis was conducted using STRING (version 11.0) (Figure 5(b)). Bioinformatics data suggests that among the 70 common target genes, the mitogen-activated protein kinase (MAPK) and phosphatidylinositol 3-kinase (PI3K)/protein kinase B (AKT) signaling pathways are most closely related (Figure 5(c)). Given that the MAPK and PI3K-PKB/Akt pathways are thought to be associated with fibrosis in many organs [18–20], we used GO analysis to enrich these two signaling pathways, which suggested an association with protein tyrosine kinase activity (Figure 5(d)). We hypothesized that the reduction of renal fibrosis by anlotinib is transduced through the MAPK and PI3K-PKB/Akt pathways. We further validated our hypothesis using Western blotting, and as expected, anlotinib significantly reduced ERK (*P* < 0.01) and AKT (*P* < 0.05) in TGF-*β*1-exposed human proximal tubular cells, as shown in Figures 5(e) and 5(f). Taken together, anlotinib reduces renal fibrosis by inhibiting ERK and AKT pathways through TGF-*β*1 signaling transduction.

## 4. Discussion

The purpose of this study was to investigate whether anlotinib could ameliorate functional and pathological renal injury in fibrotic nephropathy mouse models. Therefore, we studied the FA-induced and UUO-induced renal injury models and attempted to illustrate the potential mechanism of action of anlotinib against renal fibrosis. We first demonstrated that anlotinib improved renal function after FA-induced renal injury and reduced proteinuria and serum creatinine. Anlotinib was found to reduce renal fibrosis by significantly attenuating inflammation and matrix protein expression in UUO mouse models of FA-induced kidney injury. In addition, we also found that TGF-*β*1 expression was inhibited and decreased in mice treated with anlotinib for renal fibrosis. The results of in vitro experiments further support that the renoprotective effect of anlotinib may be mediated through the TGF-*β*1 signaling pathway.

Anlotinib is effective against a wide range of tumors, and inhibition of inflammatory and fibrotic processes in mouse models of bleomycin-induced pulmonary fibrosis has already been reported [13]. Other than that, no literature has been published regarding anlotinib's antifibrotic effects. Our study provides the first evidence that anlotinib has similar anti-inflammatory and antifibrotic effects on the kidney after FA-induced kidney injury in mice and anti-inflammatory and antifibrotic effects in the UUO models. Anlotinib has been shown to exert a nephroprotective effect.

According to previous studies, TGF-*β*1 is associated with renal fibrosis, inflammation, and progression of kidney disease [16, 21]. CKD and renal fibrosis can result from several underlying etiologies. Regardless of the cause, TGF-*β*1 is significantly upregulated both in animal experiments and in human kidney disease. Meanwhile, TGF-*β*1 has been identified as the most potent EMT inducer, inducing the conversion of renal tubular epithelial cells (TECs) to myofibroblasts [22, 23]. A number of pathways are involved in the regulation of renal fibrosis meridian [24], and the TGF-*β*1 pathway plays a crucial role among them; therefore, regulation of TGF-*β*1 expression is considered effective and important for the treatment of renal fibrosis/chronic kidney disease [25]. Recently, it has been reported that anlotinib has a profibrotic effect by inhibiting TGF-*β*1 in lung fibroblasts [13]. Inspired by their findings, we showed a significant downregulation of TGF-*β*1 in the anlotinib-treated group compared to the untreated group after establishing UUO mouse models. These studies indicated a close relationship between TGF-*β*1 overexpression and renal fibrosis. It is therefore suggested that anlotinib improves renal function and antifibrosis by inhibiting TGF-*β*1 expression.

A key function of TGF-*β*1 is to promote renal fibrosis, mainly through Smad and non-Smad signaling pathways. The Akt signaling pathway in the non-Smad pathway regulates EMT, and the activation of Akt is a key link in the induction of EMT by TGF-*β*1 [26]. In addition, extracellular signal-regulated protein kinases (ERK), one of the mitogen-activated protein kinase (MAPK) signaling pathway family, may affect renal tubular interstitial fibrosis through independent mechanisms [27]. The main mechanism is that angiotensin II (AngII) acts on the AT1 receptor in proximal tubular epithelial cells and stimulates the phosphorylation of caveolin-1 (Cav-1) and epidermal growth factor receptor (EGFR), which induces the phosphorylation of these proteins in the proximal tubular epithelium. Phosphorylated proteins cross-linked on trap protein-rich lipid membrane rafts, causing sustained EGFR- ERK signaling and promoting EMT of tubular epithelial cells. In the present study, HK-2 cells showed significantly elevated inflammatory cytokines, collagen, and *α*-SMA in response to TGF-*β*1 stimulation. Further studies suggested that EKR and AKT phosphorylation levels were significantly increased, and all of the above expressions were significantly decreased after anlotinib treatment. Our study shows that TGF-*β*1 stimulates ERK and AKT signaling channels and promotes renal tubular interstitial fibrosis, and anlotinib is used to reduce the inflammatory response, delay EMT, and improve renal fibrosis by inhibiting TGF-*β*1.

## 5. Conclusions

In conclusion, we confirmed through in vivo and in vitro experiments that anlotinib inhibits inflammatory cell infiltration and reduces inflammatory cytokines, decreases ECM accumulation, and improves renal function and tubulointerstitial fibrosis by inhibiting TGF-*β*1-mediated ERK and AKT signaling pathways. This study provides the basis for basic research related to the treatment of CKD with anlotinib.

## Data Availability

The data that support the findings of this study are available from the corresponding authors upon reasonable request.

## Supplementary Materials

Supplementary Materials Additional file 1: the primers used in this study. Additional file 2: molecular targets of anlotinib. Additional file 3: targets associated with renal fibrosis. Supplementary Figure S1: the effect of anlotinib on renal function and fibrosis phenotype in vitro. (A) Dose-dependent cytotoxicity of anlotinib in healthy mice by examining 24-hour urinary albumin excretion and serum creatinine. (B) Quantitative RT-PCR was performed to determine the RNA expression of *α*-SMA, collagen I in the kidney tissue of UUO mice treated with anlotinib in different dose. (C) Dose-dependent cytotoxicity of anlotinib in HK-2 human renal proximal tubule cells by CCK-8. (D) Human proximal tubular cells pretreated with/without anlotinib for 4 hours were incubated with TGF-*β*1 for 48 hours. Real-time RT-PCR results showed TGF-*β*1-induced *α*-SMA and collagen I mRNA expression in the presence of anlotinib with different dose (*n* = 3). Results are presented as mean ± SEM. ^∗∗^*P* < 0.01, n.s indicates not significant (*P* > 0.05), *n* = 3.
