# Supplementary material for: Anlotinib Alleviates Renal Fibrosis via Inhibition of the ERK and AKT Signaling Pathways
Source: Oxid Med Cell Longev. 2023 Feb 18;2023:1686804. doi: 10.1155/2023/1686804 (PMC9966823; doi:10.1155/2023/1686804)
Supplement: Supplementary Materials — Additional file 1: the primers used in this study. Additional file 2: molecular targets of anlotinib. Additional file 3: targets associated with renal fibrosis. Supplementary Figure S1: the effect of anlotinib on renal function and fibrosis phenotype in vitro. (A) Dose-dependent cytotoxicity of anlotinib in healthy mice by examining 24-hour urinary albumin excretion and serum creatinine. (B) Quantitative RT-PCR was performed to determine the RNA expression of α-SMA, collagen I in the kidney tissue of UUO mice treated with anlotinib in different dose. (C) Dose-dependent cytotoxicity of anlotinib in HK-2 human renal proximal tubule cells by CCK-8. (D) Human proximal tubular cells pretreated with/without anlotinib for 4 hours were incubated with TGF-β1 for 48 hours. Real-time RT-PCR results showed TGF-β1-induced α-SMA and collagen I mRNA expression in the presence of anlotinib with different dose (n = 3). Results are presented as mean ± SEM. ∗∗P < 0.01, n.s indicates not significant (P > 0.05), n = 3. [file 1686804.f1.zip › Additional file 1.docx]

| Primer name | Sequence (5’-3’) |
| --- | --- |
| mCol IV-F | ACTGGATGCCCAGGTTGTTA |
| mCol IV-R | CCTGGTGTGGAAGGACTGG |
| mCol I-F | GCTCCTCTTAGGGGCCACT |
| mCol I-R | CCACGTCTCACCATTGGGG |
| mTgfb1-F | CACGTGGAAATCAACGGGAT |
| mTgfb1-R | GCGCACAATCATGTTGGACA |
| mαSMA-F | GTTCAGTGGTGCCTCTGTCA |
| mαSMA-R | ACTGGGACGACATGGAAAAG |
| mβ-actin-F | CAGCTGAGAGGGAAATCGTG |
| mβ-actin-R | CGTTGCCAATAGTGATGACC |
| mIL-16 -F | AAGAGCCGGAAATCCACGAAA |
| mIL-16 -R | GTGCGAGGTCTGGGATATTGC |
| mCCL2-F | TAAAAACCTGGATCGGAACCAAA |
| mCCL2-R | GCATTAGCTTCAGATTTACGGGT |
| mCCL5-F | TTTGCCTACCTCTCCCTCG |
| mCCL5-R | CGACTGCAAGATTGGAGCACT |
| HCol IV-F | CGGGTACCCAGGACTCATAG |
| HCol IV-R | GGACCTGCTTCACCCTTTTC |
| HTgfb1-F | TCAGACATTCGGGAAGCAGT |
| HTgfb1-R | ACGCCAGGAATTGTTGCTAT |
| HαSMA-F | AAAAGACAGCTACGTGGGTGA |
| HαSMA-R | GCCATGTTCTATCGGGTACTTC |
| HCol I-F | GAGGGCCAAGACGAAGACATC |
| HCol I-R | CAGATCACGTCATCGCACAAC |
| HGAPDH-F | AGCCACATCGCTCAGACAC |
| HGAPDH-R | GCCCAATACGACCAAATCC |
