# Supplementary material for: Anlotinib Alleviates Renal Fibrosis via Inhibition of the ERK and AKT Signaling Pathways
Source: Oxid Med Cell Longev. 2023 Feb 18;2023:1686804. doi: 10.1155/2023/1686804 (PMC9966823; doi:10.1155/2023/1686804)
Supplement: Supplementary Materials — Additional file 1: the primers used in this study. Additional file 2: molecular targets of anlotinib. Additional file 3: targets associated with renal fibrosis. Supplementary Figure S1: the effect of anlotinib on renal function and fibrosis phenotype in vitro. (A) Dose-dependent cytotoxicity of anlotinib in healthy mice by examining 24-hour urinary albumin excretion and serum creatinine. (B) Quantitative RT-PCR was performed to determine the RNA expression of α-SMA, collagen I in the kidney tissue of UUO mice treated with anlotinib in different dose. (C) Dose-dependent cytotoxicity of anlotinib in HK-2 human renal proximal tubule cells by CCK-8. (D) Human proximal tubular cells pretreated with/without anlotinib for 4 hours were incubated with TGF-β1 for 48 hours. Real-time RT-PCR results showed TGF-β1-induced α-SMA and collagen I mRNA expression in the presence of anlotinib with different dose (n = 3). Results are presented as mean ± SEM. ∗∗P < 0.01, n.s indicates not significant (P > 0.05), n = 3. [file 1686804.f1.zip › Additional file 2.pdf]

Additional file 2

Targets of anlotinib obtained through SwissTargetPredic

Target	Common name	Uniprot ID	ChEMBL ID	Target class	Probability*	Known actives (3D/2D)
Tyrosine-protein kinase receptor UFO	AXL	P30530	CHEMBL4895	Kinase	0.1061658	Aug-72
Tyrosine-protein kinase receptor TYRO3	TYRO3	Q06418	CHEMBL5314	Kinase	0.1061658	Mar-53
Proto-oncogene tyrosine-protein kinase MER	MERTK	Q12866	CHEMBL5331	Kinase	0.1061658	Mar-46
Receptor protein-tyrosine kinase erbB-2	ERBB2	P04626	CHEMBL1824	Kinase	0.1061658	178/5
Tyrosine-protein kinase BRK	PTK6	Q13882	CHEMBL4601	Kinase	0.1061658	2月22日
Serine/threonine-protein kinase Aurora-	AURKB	Q96GD4	CHEMBL2185	Kinase	0.1061658	143/14
Vascular endothelial growth factor receptor	FLT1	P17948	CHEMBL1868	Kinase	0.1061658	79/18
Epidermal growth factor receptor erbB1	EGFR	P00533	CHEMBL203	Kinase	0.1061658	597/31
Vascular endothelial growth factor receptor	KDR	P35968	CHEMBL279	Kinase	0.1061658	485/95
Tyrosine-protein kinase SRC	SRC	P12931	CHEMBL267	Kinase	0.1061658	448/42
Hepatocyte growth factor receptor	MET	P08581	CHEMBL3717	Kinase	0.1061658	314/108
Serine/threonine-protein kinase GAK	GAK	O14976	CHEMBL4355	Kinase	0.1061658	2月16日
Kinesin-1 heavy chain/Tyrosine-protein kinase receptor RET	RET	P07949	CHEMBL2041	Kinase	0.1061658	Nov-72
ALK tyrosine kinase receptor	ALK	Q9UM73	CHEMBL4247	Kinase	0.1061658	207/3
Tyrosine-protein kinase ABL	ABL1	P00519	CHEMBL1862	Kinase	0.1061658	127/11
Stem cell growth factor receptor	KIT	P10721	CHEMBL1936	Kinase	0.1061658	106/10
Activin receptor type-1	ACVR1	Q04771	CHEMBL5903	Kinase	0.1061658	1月29日
Vascular endothelial growth factor receptor	FLT4	P35916	CHEMBL1955	Kinase	0.1061658	Aug-37
Tyrosine-protein kinase receptor FLT3	FLT3	P36888	CHEMBL1974	Kinase	0.1061658	156/14
Platelet-derived growth factor receptor alpha	PDGFRA	P16234	CHEMBL2007	Kinase	0.1061658	57/14
Fibroblast growth factor receptor 1	FGFR1	P11362	CHEMBL3650	Kinase	0.1061658	189/9
TGF-beta receptor type I	TGFBR1	P36897	CHEMBL4439	Kinase	0.1061658	Apr-78
Tyrosine-protein kinase LCK	LCK	P06239	CHEMBL258	Kinase	0.1061658	167/19
Tyrosine-protein kinase BTK	BTK	Q06187	CHEMBL5251	Kinase	0.1061658	May-67
Tyrosine-protein kinase Lyn	LYN	P07948	CHEMBL3905	Kinase	0.1061658	Jun-76
Fibroblast growth factor receptor 3	FGFR3	P22607	CHEMBL2742	Kinase	0.1061658	Mar-37

Platelet-derived growth factor receptor beta	PDGFRB	P09619	CHEMBL1913	Kinase	0.1061658	114/8
Tyrosine-protein kinase YES	YES1	P07947	CHEMBL2073	Kinase	0.1061658	Mar-34
Fibroblast growth factor receptor 2	FGFR2	P21802	CHEMBL4142	Kinase	0.1061658	35/27
Tyrosine-protein kinase FGR	FGR	P09769	CHEMBL4454	Kinase	0.1061658	4月17日
Macrophage colony stimulating factor receptor	CSF1R	P07333	CHEMBL1844	Kinase	0.1061658	149/7
Tyrosine-protein kinase BLK	BLK	P51451	CHEMBL2250	Kinase	0.1061658	4月20日
Serine/threonine-protein kinase PLK4	PLK4	O00444	CHEMBL3788	Kinase	0.1061658	2月14日
Ephrin receptor	EPHB4	P54760	CHEMBL5147	Kinase	0.1061658	3月30日
Tyrosine-protein kinase FYN	FYN	P06241	CHEMBL1841	Kinase	0.1061658	Mar-36
Dual specificity mitogen-activated protein kinase kinase 2	MAP2K2	P36507	CHEMBL2964	Kinase	0.1061658	11月3日
Tyrosine-protein kinase HCK	HCK	P08631	CHEMBL3234	Kinase	0.1061658	4月30日
Serine/threonine-protein kinase 10	STK10	O94804	CHEMBL3981	Kinase	0.1061658	4月13日
Tyrosine-protein kinase ABL2	ABL2	P42684	CHEMBL4014	Kinase	0.1061658	4月13日
Tyrosine-protein kinase TIE-2	TEK	Q02763	CHEMBL4128	Kinase	0.1061658	24/16
Ephrin type-A receptor 8	EPHA8	P29322	CHEMBL4134	Kinase	0.1061658	9月3日
Serine/threonine-protein kinase 2	SLK	Q9H2G2	CHEMBL4202	Kinase	0.1061658	4月17日
Tyrosine-protein kinase FRK	FRK	P42685	CHEMBL4223	Kinase	0.1061658	3月15日
Ephrin type-A receptor 6	EPHA6	Q9UF33	CHEMBL4526	Kinase	0.1061658	10月4日
TRAF2- and NCK-interacting kinase	TNIK	Q9UKE5	CHEMBL4527	Kinase	0.1061658	4月20日
Serine/threonine-protein kinase MST1	STK4	Q13043	CHEMBL4598	Kinase	0.1061658	11月3日
Mitogen-activated protein kinase kinase kinase kinase 5	MAP4K5	Q9Y4K4	CHEMBL4852	Kinase	0.1061658	12月4日
Casein kinase I epsilon	CSNK1E	P49674	CHEMBL4937	Kinase	0.1061658	12月3日
Ephrin type-A receptor 3	EPHA3	P29320	CHEMBL4954	Kinase	0.1061658	11月3日
Tyrosine-protein kinase receptor Tie-1	TIE1	P35590	CHEMBL5274	Kinase	0.1061658	11月4日
Mitogen-activated protein kinase kinase kinase kinase 3	MAP4K3	Q8IVH8	CHEMBL5432	Kinase	0.1061658	3月14日
Serine/threonine-protein kinase SIK2	SIK2	Q9H0K1	CHEMBL5699	Kinase	0.1061658	12月3日
Mitogen-activated protein kinase kinase kinase kinase 1	MAP4K1	Q92918	CHEMBL5749	Kinase	0.1061658	3月14日
Serine/threonine-protein kinase 33	STK33	Q9BYT3	CHEMBL6005	Kinase	0.1061658	4月16日
Mitogen-activated protein kinase kinase kinase kinase 4	MAP4K4	O95819	CHEMBL6166	Kinase	0.1061658	4月17日
Serine/threonine-protein kinase TAO2	TAOK2	Q9UL54	CHEMBL1075195	Kinase	0.1061658	7月3日
Serine/threonine-protein kinase TAO3	TAOK3	Q9H2K8	CHEMBL5701	Kinase	0.1061658	12月2日

Voltage-gated calcium channel alpha2/delta subunit 1	CACNA2D1	P54289	CHEMBL1919	Calcium channel auxiliary subunit alpha2delta	0.1061658	15/0
Voltage-gated calcium channel alpha2/delta subunit 2	CACNA2D2	Q9NY47	CHEMBL3896	Calcium channel auxiliary subunit alpha2delta	0.1061658	4/0
Receptor protein-tyrosine kinase erbB-4	ERBB4	Q15303	CHEMBL3009	Kinase	0.1061658	3月29日
Mitogen-activated protein kinase kinase kinase 8	MAP3K8	P41279	CHEMBL4899	Kinase	0.1061658	32/0
Serine/threonine-protein kinase/endoribonuclease IRE1	ERN1	O75460	CHEMBL1163101	Enzyme	0.1061658	1月18日
Macrophage-stimulating protein	MST1R	Q04912	CHEMBL2689	Kinase	0.1061658	8月11日
Serine/threonine-protein kinase Aurora-	AURKC	Q9UQB9	CHEMBL3935	Kinase	0.1061658	2月15日
Fibroblast growth factor receptor 4	FGFR4	P22455	CHEMBL3973	Kinase	0.1061658	2月15日
Ephrin type-A receptor 7	EPHA7	Q15375	CHEMBL4602	Kinase	0.1061658	7月3日
Dual specificity mitogen-activated protein kinase kinase 5	MAP2K5	Q13163	CHEMBL4948	Kinase	0.1061658	11月4日
Serine/threonine-protein kinase RIPK2	RIPK2	O43353	CHEMBL5014	Kinase	0.1061658	6月4日
Discoidin domain-containing receptor 2	DDR2	Q16832	CHEMBL5122	Kinase	0.1061658	3月13日
Activin receptor type-1B	ACVR1B	P36896	CHEMBL5310	Kinase	0.1061658	6月1日
Epithelial discoidin domain-containing receptor 1	DDR1	Q08345	CHEMBL5319	Kinase	0.1061658	4月14日
Mitogen-activated protein kinase kinase kinase kinase 2	MAP4K2	Q12851	CHEMBL5330	Kinase	0.1061658	3月15日
Misshapen-like kinase 1	MINK1	Q8N4C8	CHEMBL5518	Kinase	0.1061658	4月14日
Leukocyte tyrosine kinase receptor	LTK	P29376	CHEMBL5627	Kinase	0.1061658	10月3日
Serine/threonine-protein kinase 35	STK35	Q8TDR2	CHEMBL5651	Kinase	0.1061658	9月4日
Ephrin type-A receptor 1	EPHA1	P21709	CHEMBL5810	Kinase	0.1061658	9月3日
Ephrin receptor	EPHB6	O15197	CHEMBL5836	Unclassified protein	0.1061658	11月4日
Receptor tyrosine-protein kinase erbB-3	ERBB3	P21860	CHEMBL5838	Kinase	0.1061658	4月3日
Serine/threonine-protein kinase MST4	STK26	Q9P289	CHEMBL5941	Kinase	0.1061658	12月2日
Eukaryotic translation initiation factor 2-alpha kinase 1	EIF2AK1	Q9BQI3	CHEMBL6029	Kinase	0.1061658	3月3日
SPS1/STE20-related protein kinase YSK4	MAP3K19	Q56UN5	CHEMBL6191	Kinase	0.1061658	4月15日
Serine/threonine-protein kinase AKT2	AKT2	P31751	CHEMBL2431	Kinase	0.1061658	318/0
Protein kinase C gamma	PRKCG	P05129	CHEMBL2938	Kinase	0.1061658	38/0

Serine/threonine-protein kinase AKT	AKT3	Q9Y243	CHEMBL4816	Kinase	0.1061658	73/0
Serine/threonine-protein kinase PIM1	PIM1	P11309	CHEMBL2147	Kinase	0.1061658	638/0
Serine/threonine-protein kinase PIM2	PIM2	Q9P1W9	CHEMBL4523	Kinase	0.1061658	448/0
Serine/threonine-protein kinase PIM3	PIM3	Q86V86	CHEMBL5407	Kinase	0.1061658	331/0
Cyclin-dependent kinase 2/cyclin E1	CCNE1 CDK2	P24864 P24941	CHEMBL1907605	Kinase	0.1061658	74/0
Tyrosine-protein kinase JAK1	JAK1	P23458	CHEMBL2835	Kinase	0.1061658	137/0
Dipeptidyl peptidase VIII	DPP8	Q6V1X1	CHEMBL4657	Protease	0.1061658	346/0
Dipeptidyl peptidase IX	DPP9	Q86TI2	CHEMBL4793	Protease	0.1061658	239/0
Phosphodiesterase 4B	PDE4B	Q07343	CHEMBL275	Phosphodiesterase	0.1061658	43/0
Protein kinase C iota	PRKCI	P41743	CHEMBL2598	Kinase	0.1061658	287/0
Cyclin-dependent kinase 2	CDK2	P24941	CHEMBL301	Kinase	0.1061658	170/0
Cyclin-dependent kinase 1	CDK1	P06493	CHEMBL308	Kinase	0.1061658	146/0
Cyclin T1	CCNT1	O60563	CHEMBL2108	Other cytosolic protein	0.1061658	111/0
Telomerase reverse transcriptase	TERT	O14746	CHEMBL2916	Enzyme	0.1061658	79/0
Sodium/calcium exchanger 1	SLC8A1	P32418	CHEMBL4076	Electrochemical transporter	0.1061658	44/0
Gonadotropin-releasing hormone receptor	GNRHR	P30968	CHEMBL1855	Family A G protein-coupled receptor	0.1061658	431/0
Amine oxidase, copper containing	AOC3	Q16853	CHEMBL3437	Enzyme	0.1061658	19/0
