# Supplementary material for: Anlotinib Alleviates Renal Fibrosis via Inhibition of the ERK and AKT Signaling Pathways
Source: Oxid Med Cell Longev. 2023 Feb 18;2023:1686804. doi: 10.1155/2023/1686804 (PMC9966823; doi:10.1155/2023/1686804)
Supplement: Supplementary Materials — Additional file 1: the primers used in this study. Additional file 2: molecular targets of anlotinib. Additional file 3: targets associated with renal fibrosis. Supplementary Figure S1: the effect of anlotinib on renal function and fibrosis phenotype in vitro. (A) Dose-dependent cytotoxicity of anlotinib in healthy mice by examining 24-hour urinary albumin excretion and serum creatinine. (B) Quantitative RT-PCR was performed to determine the RNA expression of α-SMA, collagen I in the kidney tissue of UUO mice treated with anlotinib in different dose. (C) Dose-dependent cytotoxicity of anlotinib in HK-2 human renal proximal tubule cells by CCK-8. (D) Human proximal tubular cells pretreated with/without anlotinib for 4 hours were incubated with TGF-β1 for 48 hours. Real-time RT-PCR results showed TGF-β1-induced α-SMA and collagen I mRNA expression in the presence of anlotinib with different dose (n = 3). Results are presented as mean ± SEM. ∗∗P < 0.01, n.s indicates not significant (P > 0.05), n = 3. [file 1686804.f1.zip › Additional file 3.pdf]

Renal Fibrc	Targets of	Renal Fibrosis_Related genes|Targets of Anlotinib
CFTR	PTK6	TERT
HNF1B	GAK	MET
RTEL1	FGR	RET
NPHP3	EPHB4	FGFR3
TGFB1	STK10	DPP9
VHL	ABL2	EGFR
PAX2	EPHA8	PDGFRA
PKD1	FRK	PDGFRB
ACE	EPHA6	FGFR1
CEP290	TNIK	SRC
REN	MAP4K5	KIT
TMEM67	CSNK1E	ERBB2
SLC4A1	MAP4K3	FGFR2
CLCN5	SIK2	KDR
PARN	MAP4K1	TGFBR1
KIF21A	STK33	FLT1
CFTR-AS1	MAP4K4	AXL
LOC11167	TAOK2	TEK
FH	TAOK3	FLT3
NPHP1	CACNA2D	DDR1
ATP6V1B1	AURKC	FLT4
EGF	MINK1	BLK
CC2D2A	LTK	ERN1
RPGRIP1L	STK35	MST1R
MTOR	STK26	JAK1
SLC4A4	MAP3K19	CSF1R
FLCN	PRKCG	MAP2K2
NPHP4	PIM2	AKT3
GATA3	PIM3	ERBB4
PKHD1	DPP8	CDK2
LOC110806263		MERTK
TP53		DDR2
SFTPC		LYN
SLC2A9		ALK
AGT		ERBB3
TERC		CDK1
NPHP3-ACAD11		SLK
PKD2		AKT2
MUC1		EPHA3
LOC111674475		TIE1
HNF1A		ABL1
AGTR1		FYN
NEK8		AOC3
WDR19		YES1
TTC21B		SLC8A1
SLC22A12		FGFR4
INVS		ACVR1
TSC1		HCK
ATM		MAP3K8
UMOD		MAP4K2
CCN2		ACVR1B
ATP6V0A4		PDE4B
HFE		PLK4
SETD2		PIM1
TNF		LCK
LOC111674477		AURKB
IL6		GNRHR

WT1
HMOX1
ALB
CLDN16
SERPINA1
VPS33B
SLC5A2
IQCB1
MKS1
SLC22A5
TMEM216
IFT140
LOC113664106
INPP5E
TUBB3
SDCCAG8
PHOX2A
IFNG
B2M
CLDN19
VEGFA
ABCA3
IFT172
ITGA8
FHIT
DSP
EDNRA
FXYD2
CAV1
CTNNB1
TSC2
SLC9A3
AQP2
AHI1
OGG1
CA2
MITF
NPHS1
VIPAS39
UPK3A
FN1
TMEM231
BSND
FAM13A
SCNN1A
RRM2B
INS
TMEM237
FCGR2A
COL4A5
HLA-DRB1
LOC113633877
SLC34A1
EP300
PTEN
MIR21
SCARB2
IL10
RIPK2
BTK
EIF2AK1
PRKCI
CCNE1
TYRO3
EPHA1
CACNA2D1
EPHB6
MAP2K5
STK4
EPHA7
CCNT1

PIK3CA
SCNN1G
STN1
TCTN2
B9D2
STX1A
EDN1
EPO
MIR141
CRP
COL4A4
SIX1
MIF
OFD1
B9D1
CSPP1
CDH1
HIF1A
CCL2
DYNC2H1
BICC1
FGF23
SERPINE1
MMP9
BRAF
TRAF3IP1
APOL1
APOA1
FRAS1
OCRL
CXCL8
TCTN3
MIR200C
SPP1
SCNN1B
HGF
IL1B
COL4A3
WDR35
TMCO1
CEACAM3
KCNN4
TCTN1
TFEB
NPPA
CCND1
IL2
DCDC2
SARS2
RFH1
BMP4
LYZ
SLC26A9
IFT122
IL4
CASR
AKT1
SLC6A14

NPHP3-AS1
STAT3
MMP2
CLCNKB
STAT1
SDHB
MKKS
DNAJB11
GSTM3
NPHS2
IL1RN
CEP164
SLC9A3R1
NR3C2
THBD
SLC12A3
FOXI1
SLC11A1
GSN
AVPR2
C12orf29
HPS5
ANKS6
GCLC
TIMP1
KL
NDUFAF6
LMX1B
ARL3
C3
IFT80
HPS6
NONO
MMP1
TMEM138
SMAD4
TLR4
ABCB1
CFH
ICAM1
CEACAM6
CCR6
SLC12A1
MME
KCNJ1
KRT18
CLCA4
CEP120
NFE2L2
NLRP3
IFT43
TTC21B-AS1
ENG
HPS1
LOC105371046
BBS10
GLIS2
BMP6

FGA
IGF1
ALMS1
FAS
HS2ST1
ELN
KIF7
NOS3
FASLG
CP
KRT7
LRP2
INF2
GATM
VIM
ZNF423
KIAA0586
DZIP1L
ARL13B
APOE
KIAA1109
XPNPEP3
LCN2
BBS4
CLTC
FGF2
IL13
LDHA
CD2AP
ACTN4
CPLANE1
TMEM260
BBS2
GNAS
PBX1
HNF4A
NAGLU
KYNU
ABCB4
RMND1
CEP83
WDR72
BBS1
IFNA1
IGF2
NEK1
CSF2
BRCA2
CDKN2A
SMAD3
MT-TL1
CD36
PPARG
CST3
NHLRC2
HPS3
IL18
IRF5

SALL4
CEP41
EHHADH
MEFV
BGLAP
H19
NPPB
BBS5
GLA
JUN
VDR
SUFU
CTLA4
SEC61A1
PHEX
MPO
CXCR3
APC
ABCB11
BMP7
JAG1
PRTN3
POLE
AQP1
PRKAR1A
PTGS2
MMP7
GREM1
NHP2
MDM2
HPS4
IGFBP3
BBS12
GEMIN4
RPGRIP1
ALG9
ELANE
DIRC1
CUBN
CA9
NUP107
TTR
KAT6B
GANAB
SOX18
TUBB2B
TUBA1A
PAX8
TTC8
CALR
MYH9
TBX18
CDC73
CFHR5
SMAD2
TTC26
FBN1
IFT27

TINF2
TRPC6
COL4A1
SLC34A3
HRAS
BCS1L
DNASE1
LOX
RBP4
GPC3
SLC7A7
HSP90AA1
CTNS
TMEM107
MAPKBP1
BBS7
GSTP1
CCL5
MIR199A1
IL2RA
BBS9
AGTR2
TMEM127
TXNDC15
IL17A
CDKN1B
CHD7
TNFRSF1A
ACTB
VPS45
CLCN1
SMAD7
IFNA2
APRT
SFTPD
CLCNKA
CXCL10
GGT1
WNT4
CD4
S100A1
SLC2A2
PTH
LGALS3
PTPRO
CFHR1
EBF3
CEP104
AGXT
DIS3L2
SGK1
DTNBP1
DYNC2I1
TRIM28
MBL2
SMARCAL1
ACP5
SPINK1

MIR155
KCNJ11
NOTCH2
CFI
EVC2
FAN1
ENPP1
HLA-B
VCAM1
IFT52
GLIS3
SLPI
APOB
DYNC2I2
IL1A
KRT19
GPT
TLR2
MIR192
ADIPOQ
EPAS1
IL5
F2
SHH
KRT8
ITGA3
KRAS
TF
PTCH1
CASP3
SNAI1
C2CD3
BAP1
NRIP1
JAK2
NOP10
FANCD2
DEFB1
DKC1
NOS2
XDH
GLI3
HP
SDHC
HPRT1
COL1A1
NOX4
NUP133
BCL2
CCL3
SLC22A6
ROBO1
SLC26A4
COQ8B
MYO5B
LAMB2
SLSN3
MSH2

SDHA
CRB2
STAR
LEP
NKX2-1
AGER
HLA-A
EPCAM
MIR17
NRAS
AMBP
HAMP
S100A8
MAPK1
C12orf60
ATP8B1
F3
CD274
SLC3A1
CD80
TRPV4
PDCD1
HBB
IL1R1
MFF-DT
GSTM1
KNG1
PDPN
KIAA0753
CYP11B2
MIR30A
CXCR4
GRHPR
CXCL9
MIR34A
PDGFB
PLG
PIK3C2A
PRKCD
CSF3
ADCY10
ABCC8
PIBF1
PEX1
FGF10
CPT2
SLC2A1
MAPK3
CA3-AS1
MYC
ARL6
NF1
CA12
HSPG2
VPS33A
LCAT
KCNQ1
NOTCH1

SLIT2
ZMYM2
APOA1-AS
IGFBP5
LMNA
TJP2
SOX9
SDHD
CDKN3
PMM2
FOXP3
TGFA
FAM20C
CALCA
ZEB2
MIR140
TP63
ADRB2
PON1
CD46
AQP3
FGF8
ANXA2
TIMP2
KATNIP
HMGB1
MAGI2
CCL11
S100A9
ELOC
COQ2
PEX2
CDH16
MAPK8
TGFB2
CA4
MIR126
PMS2
HNFJ3
SLC6A19
MT-ATP6
MMACHC
PDE6D
FAH
MMP3
BRCA1
PRKCSH
CHI3L1
MTHFR
EZH2
SEC63
CASP8
MIR15A
TOGARAM1
MUC5AC
PVT1
SLC17A5
ABCG2

IFT74
BBIP1
NR1H4
GAPDH
CD34
ITGAM
LZTFL1
CD8A
NUP93
CD44
PAX6
MIR93
ARMC9
PEX6
SMARCA4
ANGPT2
TRIM32
WDPCP
THBS1
SOD2
TGFBR2
SLC40A1
HAVCR1
KAAG1
MIR106B
SLC37A4
TMEM218
ALOX5
CFHR2
PRSS1
HSD11B2
IL33
TNFRSF11B
CDH2
GC
PEX5
SFTPB
CD40LG
COL1A2
MIR214
TET2
FANCB
MPL
MIR204
SIRT1
YAP1
HOXD13
ANOS1
GCK
PTPN22
KCTD1
ADAMTS13
ABCC1
ACE2
SERPINH1
PTPN11
VEGFC
HYLS1

CSF1
ADM
PHKG2
PEX10
TOLLIP
ATP6V1B2
PRSS8
MYO1E
DACT1
ANLN
HFE-AS1
RARB
MIR145
EVC
TNFSF10
ITGB1
RAB11A
SOD1
TFAP2A
KCNJ10
CCL4
RAF1
BIRC5
IL15
HLA-G
SLC12A2
FUZ
BLOC1S5
HOGA1
AFP
KCNQ1OT1
MIR200B
MLH1
HSPA5
STK11
TNXB
GBA
ARHGDIA
CXCL12
CFAP418
DHCR7
MTMR10
TGM2
TGFB3
WRAP53
SOX17
MEG3
RYR1
GUCY2C
SREBF1
PDGFA
PDX1
VWF
DCHS1
MIR29C
WNT5A
EZR
ARG1

PEX3
CD79A
FBXW7
RHOA
RAD51C
PLCE1
APOA2
MALAT1
DYNC2LI1
COL3A1
COPA
FANCC
CXCR2
SLC25A13
DUSP29
SIX2
SERPINC1
ATP12A
MIR142
MIR221
MEN1
SPRY2
IL12A
HSPA4
MYO5A
NOD2
PROKR2
BCL2L1
FGF7
ADORA1
MIR27A
MPI
KANK1
CCR5
CD40
CEP19
LIPA
HJV
CBY1
PTPRC
TALDO1
PTHLH
ACTC1
LAMA3
BSG
IGF1R
NEUROD1
RNASE3
DNMT1
PECAM1
G6PC1
WFS1
PEX19
TRPM6
SLC9A3R2
CR1
GDNF
MKI67

DICER1
IL9
GUSB
LBR
MIR122
COQ6
TRIM8
GPC4
HLA-DPB1
ANXA5
PHKA2
FAT4
BTNL2
MSH6
TYMP
CCL17
FANCI
KEAP1
SLC6A20
BLOC1S5-TXNDC5
EEF1E1-BLOC1S5
HSPA8
MMP8
CXCL2
PVALB
MASP2
CXCL1
CYP1B1
MIR200A
RPL5
SPARC
HOTAIR
LARS1
MIR143
CLU
CLDN10
EPOR
MYOD1
HSPB1
DAAM2
KIRREL1
CYP1A1
CLDN7
RHEB
CCL18
DAB2
OSGEP
IL13RA2
LOC107548112
TRIM59-IFT80
BAX
ABCC2
GRP
NDUFS7
SELE
NUP85
CDKN1C
SETBP1

GNA11
SURF1
KRT20
CYP27B1
RAD51
RB1
SELP
CLCN4
RASSF1
TLR9
KLF11
MT-CO3
BRIP1
MICALL2
CCR2
PEX13
TNNT2
ACTA2
GALNT3
MT-ND4
TBC1D8B
CHUK
RBSN
POSTN
PARP1
SAA1
RPL36A-HNRNPH2
ALG8
SH2B3
CXCR1
PRKCA
MIR222
DNAJB1
MT-CYB
NUP205
LRP5
MYCN
GZMB
DDIT3
CYP3A5
SLC5A1
MT-ND5
DLL4
H2AC18
SLC34A2
CD86
RUSF1
WNK4
NOS1
DVL1
SUCLA2
FANCA
MIR210
STAT4
SLX4
AP3B1
PCNA
BMPR2

MIR26A1
SLC4A7
DNAH8
MT-ND6
ATRX
ACVRL1
CLCA1
LTF
ADORA2B
SGPL1
MUC4
FIBP
PROK2
HSPD1
MAD2L2
KIF1B
CCL22
C4A
TNFSF11
TNFRSF10B
ATF6
GATA1
LRRC56
WDR73
ATP1A1
HAVCR2
PNPLA6
CFB
CASP9
MIR196A1
UTP4
EGLN3
CLCN7
ITGB4
LDLR
TOP1
VIP
PIEZO2
FAM149B1
LACTB
MIR23B
NPM1
EIF2AK3
CLCN3
TLR5
SF3B2
BRD4
MAX
SCGB1A1
ELOB
CDKN1A
EMP2
FABP1
ANTXR1
MIR181A1
PCSK9
TUG1
KIAA0319L

HS6ST1
EDNRB
MIR150
MIR185
POU6F2
CANX
PLEC
NSD1
LAGE3
GPR35
ZIC3
PRL
IFT88
SYNPO
DERL1
NUP160
IARS1
MIR223
SP110
SRGAP1
MIR195
INSL6
ADA
ACD
S100A4
NOS1AP
MUC6
RAB5A
CFLAR
SDHAF2
SLC26A3
ALAD
SPTAN1
BMP2
TGM1
UQCRB
PAX4
MRE11
MBTPS2
DES
INSR
GATAD1
MAPK14
SLC30A7
ALDOA
GAL3ST1
STUB1
MMP12
GJB2
PLCH2
ELAVL1
RGCC
XIAP
CDK4
APEX1
PDZK1
MIR1225
ALDOB

SEMA4D
SST
MIP
KIF24
BAAT
CUL3
NAT2
SOCS1
PPARA
NF2
LIFR
TNNI3
PTX3
ENPEP
ARHGAP24
TXN
INTU
MARS1
PNPLA3
REST
MSLN
ILK
KANSL1
GPD1
GLI1
BPI
MIR144
ANPEP
ABCG5
CAT
PEX16
SFRP1
ARAF
MST1
POLG
MT-ND1
MT-CO1
PFKFB3
APPL1
BMP1
ICOSLG
ABCD3
PRKACA
RETN
NFKB1
GBE1
RPS27A
IGFBP1
PEX14
PSMB8
XRCC2
UQCC2
CAMP
NBEAL2
TIMP3
MIR22
DNAJC5
ERCC6

NIPBL
NR5A1
PEX26
PEX12
MIR29A
KIF3A
CCR7
FADD
CYP3A4
CXCL5
KDM4C
GSTT1
PTGS1
SLC17A1
C12orf57
TCF4
GAS5
LOC106501713
DYNLT2B
DCN
ABCC4
PLIN2
PGF
GNRH1
IDO1
PKM
IL3
VCP
ENPP2
STAT5B
ENO2
FANCM
F5
NBAS
LOXL2
AVP
SF3B1
GATA4
ADA2
HDAC8
IL17F
NPY
MIR224
CHGA
XBP1
KIF3B
CCR1
MT-ND3
SMPD1
FANCG
TPRKB
MIR18A
MRPS7
CYP1A2
MIR29B1
ESR1
RBPJ
AHSG

CCR4
PATJ
KRT13
HLA-DQA1
ABCC6
IFRD1
DPP4
PSMB9
MIR101-1
CTSG
HLA-DQB1
HPSE
HMGA2
MIR494
YY1AP1
VDAC1
LPAR1
MIR19A
SOST
PLAT
HOXA13
ENTPD1
KLHL3
MLXIPL
FARSB
CCNB1
ASTN2
SPINT2
RAB8A
TTC37
CHRNA3
HSD3B7
LPA
HIF1AN
NLRC4
IL2RB
VEGFD
ARSB
THPO
HDAC2
TYR
CALB2
TGIF1
ANXA1
WNT11
BLOC1S3
PARK7
GLI2
TFAP2B
LPL
SERPINA3
GUCA2A
BDKRB2
EMX2
WNT9B
ANO1
MT-TK
SKIV2L

PSMD12
CAPN10
MT-ND2
P2RY1
EPHX1
HMGCR
CCDC28B
SKIL
NTS
LEPR
AKAP13
BCR
CLTRN
SPHK1
LIMK1
APOC3
DLST
CACNG2-DT
TTN
YWHAE
GJB1
MB
TP53RK
U2AF1
MIR182
MIR31
STC1
MAGI2-AS3
WNT3
TBX1
TNFSF15
ROR2
CHN1
SP1
COL4A6
MIRLET7D
RASGRP1
WNK3
USH2A
MSR1
MIR25
MIR212
PRKAG2
ADCY6
PSMC4
HSPH1
SLC12A7
GRK2
STAT6
NCF1
MIRLET7A1
PLAU
FOS
LIG4
ADGRG2
GOPC
NR3C1
MDH2

COMT
CEL
CDC42
PODXL
PRKG1
EIF4EBP1
HLA-DPA1
RAB11B
TAP1
MMUT
MT-TE
KITLG
MAP2K1
MIR328
MIR138-1
ASL
SLC22A8
NR0B1
OSTM1
CLCN2
ARFGEF1
THG1L
BMPR1A
NKX2-5
SCN5A
DVL3
MIR9-1
RNU4ATAC
YY1
CASP10
SERPINB1
MIR34C
EPHA2
AHSA1
PRODH
KDM6A
PITX2
ZDHHC24
AP1S1
PSMD4
FURIN
IFT57
EDN3
KANK2
IL12RB1
STRA6
TNFRSF1B
DEFB4A
AQP4
COL7A1
THY1
MUC3A
KLK1
AURKA
GTF2I
C11orf65
CTAG1B
TRAP1

TREX1
LBP
B3GAT1
RBX1
BIRC7
MIR193A
EGR1
PACS1
NDUFS4
WNK1
DLL1
WNT7B
MIR130A
FOLH1
TKT
PAH
ITGA6
UCA1
DKK1
PSMC6
MT-CO2
TJP1
CTSK
AR
PCK1
FIP1L1
GALK1
ASPM
CRNDE
GHRL
CHRM3
MUTYH
LAMP2
LAMB3
SCO2
RAB7A
MGP
ERLIN2
BLM
SEMA3A
WDR4
DNMT3A
CLDN1
POR
TUBB
CRYAA
ADD1
CD70
PTRH2
NNMT
CYP11A1
FLNA
BIRC3
KMT2D
MIR15B
ANXA6
CD28
UBC

TNPO3
COG6
LNX1
PSMC5
SLC26A6
LNCARSR
CLDN4
LAMC2
NEK9
SNCA
MIR146A
CSK
SLC25A11
CRYAB
PSMB3
SP140
P2RY2
MIR99A
PSMA5
TUBB2A
IFT20
RNF5
TFRC
SLC4A5
LPO
PSMA1
ALDH2
HSD17B4
STX16
LINC00963
MIR135A1
SPPL2C
NEU1
BLOC1S1
MT-TW
MIR590
CCN4
RFC2
ARNT
TLR1
CYCS
GFRA1
PSMD1
AKR1B1
MIR139
CASP1
SART1
CD247
PTGDS
DMBT1
UTS2
FOXC1
SLC41A1
EGLN1
MGME1
ATP7B
MIR99B
TRRAP

PEX11B
TUBB1
ACTG2
RAD21
CELA3B
MIR181A2
COL2A1
PSMD2
HSPA1A
VCL
GAS6
SLC51B
PIK3CG
ADAMTS9-AS2
PSMB1
F2R
SHC1
DGUOK
PROM1
TMEM213
PIGY
MIR149
DYRK1A
WWTR1
MIR10B
PSMD11
CASC2
MIR423
PSMB4
TFR2
PYGL
APCS
NEDD4L
SHROOM3
APOA4
AXIN2
PSMD8
RPS19
PSMA3
MIR424
ITGB3
CTNNA1
COL14A1
AQP5
ERLIN1
GH1
F2RL1
BARD1
DGCR5
MMP14
CD151
KIF17
MYH11
RPGR
REG3A
ZNF592
ABCF2
MIR490

ATP6AP1
IFIH1
ACADVL
PTH1R
MIRLET7G
BDKRB1
LTA
PSMA6
SLC22A11
MIR296
MYRF
ATP4A
MYH10
SERPINF2
SPECC1L
AAGAB
GAPVD1
XRCC1
TPMT
MASP1
PYGM
TPBG
LUCAT1
ACOX2
SLC4A2
PSMA8
POMC
MIR532
DIPK1A
CREB1
CNR1
NAA10
SEC23B
GTF2IRD1
BAZ1B
FLII
NPPC
CCAT1
PSMA4
ALPL
MIR335
FKBP6
TCIRG1
SLC13A2
CD82
PSMA2
TULP3
MIR338
PSMB2
EIF4H
PSMC3
NOTCH3
BCL7B
TRB
IFNL3
PSMC2
ROCK1
LINC-ROR

KARS1
PSMA7
AP2S1
PANDAR
VANGL1
MIR320A
EXT2
MEPE
NDUFS2
CYP7B1
UBD
USP9X
SCT
PSMD3
ZNF148
MTX2
C5
GTF2IRD2
METTL27
PDZD3
CD163
TWIST1
SOD3
ABCG8
PSMD7
PSMD14
SPRY4-IT1
LINC00473
PTK2
NR1I2
RCBTB1
SOCS3
FOXE3
TNC
CD14
NCAPG2
NPNT
MIR885
PRKG2
STING1
DERL2
CCL26
IL9R
CYTOR
ANKFY1
HSPE1
CYS1
MECP2
PLA2G2A
MYD88
MRPS22
TNFSF13B
FTX
PSMB5
MYLK
PSME2
MRC1
SDC1

CETP
CCAT2
SCAPER
PCBD1
CPT1A
UCP2
PGR
IGF2R
PC
RAB7B
PI3
PLAUR
GNB1
ACSL4
BPIFB1
ETFDH
CLEC7A
PTENP1
CYP2E1
EWSR1
SIGLEC5
C1QA
APOH
BUD23
SRY
HABP2
ANO5
ARX
TBL2
CLIP2
LPIN1
DNASE1L3
FGF1
KCNK3
ONECUT1
MIR211
AKR1D1
BUB1B
LAMP1
CCN3
KCNJ2
TUBG1
EPX
ATD
PRR5
GJC1
AMMECR1
LYST
CREBBP
PRKCB
RHO
GPER1
DANCR
MIR377
AGK
COG1
MT-TF
NUP37

SSBP1
RPS6KB1
SLC7A11
ARHGAP31
FCGR2B
VTN
ETFA
KRT14
LEPQTL1
GDF15
MIR20A
ERCC1
UNC45A
CDH5
IL4R
GDF11
CCP110
TRPM7
WASHC5
CX3CR1
FLI1
IGFBP2
STXBP2
FSHR
PIEZO1
SYP
VPS37D
ERAP1
MIR203A
DMD
SFRP4
MMAA
BDNF
G6PC3
PRKD1
MED12
UBE2J1
CTC1
TTBK2
BNIP3
SLC26A8
PPARGC1A
DEFB103B
PRDX1
GJA1
ETFB
MAPK10
ADORA2A
FOXD1
WDR62
DNAJC30
GCG
IGHE
NPTN-IT1
C4B
CEP152
MTTP
INPP5B

FOXE1
CXCL16
DCT
TMEM270
CEBPB
POLD1
NOTCH4
IL7
GAST
AIP
LRRC37A2
ALG1
MIR708
UBE3A
NR1I3
MYH6
STS
KCNJ5
SKI
FOXF1
CYP11B1
LOC107133510
LIPC
TAC1
SCN1B
KRTCAP3
COL5A1
YWHAQ
USF2
ASAH1
FKRP
AXIN1
PRG2
SLC30A9
PLOD2
TWNK
CDKN2B
MYOCD
ANGPT1
ZNF699
TRIM37
UQCRQ
CNTLN
FIG4
SFTA3
VAC14
LOC106099062
GFAP
ABCB7
ELP1
CYP19A1
EDN2
CDH23
STK39
CADM1
MIR30E
TMSB4X
UCHL1

SLC51A
GSR
ITGAV
DEAF1
PF4
JPH2
MIR519D
CD55
CEP97
PHGDH
POT1
BLOC1S6
CCL7
CLDN3
TMEM199
FAP
FTO
STX3
AGL
ADAM19
WRN
MAP3K7
FLRT3
LECT2
OXT
MIR125A
SQSTM1
TNFRSF6B
COLEC11
IL11
FKTN
SCTR
GALE
PGK1
CHIT1
C1QBP
ESCO2
TRIM21
MAPKAP1
POU2AF1
AP3D1
IGFBP4
FOXM1
CDK6
ACTN2
EPG5
CDK5RAP3
HLA-C
LOC106627981
FCGR3B
TLR3
RAC1
RUNX2
CYP21A2
COG7
RCN1
SLC15A2
SLC29A3

PXN
EFEMP1
CTSL
MIR216B
GPX3
PIK3CD
KCNE5
KMT2C
ZFPM2
DRAIC
MED13L
PRKCZ
GON7
SCLT1
YTHDF2
F13A1
F10
UROD
RTTN
NRP1
C5AR1
POMT1
MIR378A
PCAT29
COPB2
SMC1A
XRCC3
KRT5
UQCRC2
TGFBR3
RCC2
ETV6
KCNA1
SLC13A1
ADAMTSL1
IDH1
XRCC4
TNFAIP3
SLC35D3
MAGEA4
AIM2
NEDD9
MIOX
MPV17
APEH
PIGT
SPRY1
ETS2
CAD
ASS1
ZIC2
IL6R
IL6ST
IFT46
SEC24C
RPE65
MYL9
SAGE1

CTSD
PAX7
IL12B
NARS2
MIR483
DLC1
SIX3
APOC2
KCNJ13
TUBB4B
RAD50
CYP2D6
TUBB6
FOXO1
WFDC2
LMOD1
OCLN
CD63
TCF7L2
FGF21
NME1
DHCR24
XYLT1
UGT1A1
MAP1B
TBX3
CYP2C9
SMARCC2
RECQL4
IFNGR1
MT-TS1
CMA1
IL7R
CDK5RAP2
TAP2
PTGER4
NT5E
DDRGK1
LGALS9
PLXNC1
SMC3
LOC110006319
DPM1
PLIN1
CD209
DOCK6
HDAC4
MAGEA3
ROBO3
IFT81
SERPINA7
CEP63
PRF1
IRX2
ATP6V0A2
TDGF1
HDAC9
WDR81

LTBP4
MIR191
ADK
KCNAB2
TLR7
SOX11
GCDH
ADAMTS9
ENO1
MIR1202
COL4A2
DRD2
PIGO
VPS16
TFF1
RPL35A
GATA2
RPL3L
ESR2
AARS1
LEMD3
TOE1
GHR
RPL11
RNU7-1
PHYH
DUSP6
RPL15
MCL1
CDH11
ABCC9
RARS1
KIF20A
TTC19
IMPDH1
LORICRIN
GSK3B
MRTFA
CD9
FLNB
RAB3GAP1
ARSA
UBAC2
SEMA3E
MIR342
ITGB2
PSC
SS3
RELA
RPS26
JAK3
NEAT1
CLPB
NFKBIA
CENPJ
SLC12A4
EMC10
RPL26

CACNA1S
STXBP1
NR0B2
TM6SF2
SI
ABCB6
TXNRD2
CCR3
PCK2
TNFSF12
HCN4
MIR148A
CBS
LAMA5
CD59
ITGA2
MLKL
POU5F1
HDAC1
PNPLA2
G6PD
BCAR1
ABCA1
FREM3
ARL13A
PPP2R1A
RREB1
SLC22A18
UFD1
MYL3
IGBP1
B4GAT1
KLRC4
FCGR3A
TPR
JMJD1C
PLK1
IL1RAPL1
GAS1
KIF1A
WNT3A
FTH1
SOX3
RUNX1
GP1BA
CNDP1
SELL
CSF2RA
MX1
NODAL
HSPA9
DNAJA2
OXA1L
VPS39
SSR4
BMP3
PKD1L1
XIST

TRPM3
ALG2
SAA4
MVK
IRS1
IDH2
C1QB
KLF15
TRIP11
PHETA1
ADRB1
POLG2
IAPP
ECI2
STAT5A
KIF12
MED6
HAX1
IRF1
NPR1
ADAM17
UFM1
SUCLG1
INHBA
RINT1
HSD3B1
DHDDS
STX11
SOX4
NTRK3
UNC13D
PRSS2
F8
ARHGAP1
MMP13
NDUFA1
CEACAM5
SNAI2
PGM1
IL16
UFL1
MIR451A
RXRA
BGN
RAC2
NCAM1
ADAM10
SERPINB7
NR1H2
CYBC1
MCM5
OTC
WHRN
VPS18
SASS6
FBF1
POGZ
IL1RAPL2

IL1RL1
LTBP1
CXCL13
PLA2G7
PRDM16
CXADR
HMBS
MAPT
PGAP2
KLKB1
ESRRG
CYP2C19
SLC15A1
AP1M1
ATXN10
TTC30B
SPATA5L1
NTRK1
FMN1
USP53
PEX7
RPL8
RPS10
MIR32
FBN2
HRH2
GLUD1
C1QC
CDON
KCNK5
CD69
STRN
BECN1
MICA
ZEB1
HADHB
MIR27B
MIR127
DNAJB6
TUBB4A
ITGA4
ATP8A2
RPS24
STIL
CEP135
SLCO2A1
VPS11
CDKN2B-AS1
HADHA
SGSH
CD81
ALDH18A1
GUCY2D
HSPA1B
CEBPA
POMT2
STAT2
SACM1L

ITGA2B
BCHE
ETS1
UBR1
PLA2G6
MDK
ACKR3
MIR146B
ABHD11
SH2B1
CTCF
PDGFRL
LEFTY2
FOXH1
CXCL11
PPP3CA
CC2D2B
TGFBRAP1
PGAM2
ABCA12
FASN
KIF16B
KIRREL2
TYMS
HSD3B2
CCK
IRAK1
TRPV6
TNFRSF10A
DDX58
TUBA8
DNAH11
ACTG1
SLC9A1
EPHB2
DCXR
FLNC
EXOC5
CLDN14
MRPS34
IL17RA
TH2LCRR
TH2-LCR
DNAH5
AGRN
CORIN
ALPP
AMH
CEP89
PRKDC
PXDN
S100A12
TCN2
RAB3IP
LMBRD1
RPLP2
CFP
CHD4

CABIN1
IFNB1
VPS8
MYH14
CCN1
ASXL1
LEF1
AP1M2
LDHB
INHA
COG2
SRF
EOGT
EMP1
PSAP
ORAI1
HBEGF
FUT4
MINPP1
CCBE1
EIF2AK4
KIZ
NFAT5
MUC2
SLC4A11
SLC22A4
COMP
LGALS1
MYH7
IFT22
TSLP
ITGA5
RNF168
KRT33A
VANGL2
NDUFA13
WNT1
CFL1
SERPINF1
PFAS
ABCD1
IRAK4
HDAC6
FABP4
LARGE1
CCT8
TRPV5
CPOX
PRKAA2
NXPH4
PPBP
SSTR3
HYDIN
UGCG
EFTUD2
DDAH2
CCDC115
FOXP1

GP9
CYBA
ATP6AP2
SKP2
RAB27A
SATB2
LMNB2
PCNT
TACO1
SCN4A
LIMS1
THBS2
VSTM4
NPC1
CTRC
TRMU
NID1
PTPN12
IARS2
OXTR
HPX
STK24
DLK1
TULP1
SIRT3
IKBKG
NQO1
EGLN2
KIF5C
SPATA7
FBLN5
SLC35A2
CTSB
ATF2
FOXO3
WT1-AS
MIR23A
BAK1
SHOX
PLOD1
MTHFD1
PDGFD
ATF3
IGF2BP3
IDUA
MAPK9
MT-TH
RSPO2
NGF
MORN1
CILK1
PRKN
KLF6
PLA2R1
GPBAR1
ABCC3
RPS17
FGF9

MSTN
STIP1
KLF4
GAREM1
EIF2S1
ECE1
TBX5
FABP2
SLC10A2
FSTL1
DNAI1
SLC22A1
PHB1
XCL1
TREM1
S100B
SLC25A22
GOLM1
RPS29
PPA2
ATP7A
PLD1
FCN3
ITPR3
GFER
HGD
ANKS3
STAC3
KCNMA1
CYP27A1
TBX4
RAB6A
RBM48
ITGA1
IL23R
RACK1
SBDS
WAS
GAA
MAF
CCL20
PYURF
PHKB
CRX
KCNMB1
STX4
BMP15
PRMT1
SCD
OGDH
TBXAS1
NNT
USH1C
ST13
RPS6
LINC00336
DACH1
LINC01194

SRSF2
POC1B
ADAR
RELN
ATG5
GJA5
ALOX12B
NANOG
PLA2G4A
RARA
SULT1E1
EHMT2
CACNA1A
SCARB1
CDKN2C
PITRM1
C3AR1
PTPRJ
SHBG
SLC3A2
NCOA3
LMNB1
CYP2R1
DROSHA
BCL2L11
AKR1A1
SOSTDC1
MUC16
PAPPA
PDGFC
HAS2
JUP
FBL
INSL3
APLN
RASA1
LDB3
REV3L
FGF19
UNC119
TG
CANT1
PRKAA1
MPDU1
GNB2
CRB1
DAG1
SLC10A1
ID1
EYA2
RO60
MIR133B
GPI
SREBF2
BICD2
OGA
TTC7A
RDH12

KLRK1
CD58
NAMPT
OSM
SLC5A6
ROCK2
MIR130B
TRNT1
BLMH
TREH
AIFM1
LRAT
RPTOR
CXCR5
NCAPD3
CIB3
BRAP
ZSCAN21
CPB2
P2RX7
XYLT2
YWHAB
EXOC4
CCNA2
VAMP7
MUC7
AOC1
SLC26A11
MIR148B
ERCC2
EBP
NGFR
IL22
AIPL1
DRD3
TGFBI
MYO7A
MIR181C
CX3CL1
PSMD9
SLC1A5
PDE4D
SOS1
UGT1A9
NDUFS1
TCF3
MIR196A2
MT2A
LAMB1
HBA1
PLXND1
BNC1
DRD1
AFF3
LCA5
TBCK
OAT
SOAT2

DYM
MIR19B1
TMEM17
PDS5A
GLS
MEP1B
PPP2R5C
F9
FBLN1
CGA
UNC119B
TOP2A
PHLDB2
RAG1
ITGAL
ADCY3
LAT2
NR1H3
RBM15
EPS8
MYB
MLN
LYRM7
DDAH1
COQ4
PON2
UPK1B
MIR215
ACHE
CAMK2G
CYP7A1
FOXJ1
MSN
HIF3A
YARS1
PHF11
TINAG
PPIG
GLO1
CD68
RBCK1
AK3
USP10
GYS1
TAGLN
STX8
IKZF1
SCARF1
HNMT
MT-ATP8
CCDC92
FSCN1
DYNLL1
ALPK3
COX5A
KCNJ18
MID1
NSUN2

RPS27
MFN2
TIAM1
MX2
CD27
SEC61B
MAP1LC3A
PI4K2A
MC2R
CFD
MIR154
SEMA4A
YBX1
PHF21A
TNFRSF12A
PGM3
NUB1
NOX1
SMS
GDF2
FCN2
PDE4A
RPS15A
IGF2BP2
MIR30C1
KRT10
LAMA2
PDP1
GALNS
PTGER2
CPM
ZAP70
BCL2A1
CHAT
TMEM51-AS1
BCL6
CDK5
CRHR1
EXOC1
SLC12A6
GUCA2B
SLC46A1
SIK1
MRPL3
BMP5
MYOG
AP1B1
TNFRSF13B
MIR106A
SOCS6
RBM5
CDC25A
RAMP3
CACNA1B
FTL
CTH
TRMT10C
LGALS3BP

DKK3
RUNX3
MAP2K3
TPO
SLC16A1
ASIC1
HHEX
LARS2
MAPK7
SLC28A1
ADGRG1
MIR199B
GPX1
NKX6-2
HYAL2
ALX4
AHR
PTGER1
JAG2
SLC7A2
RIPK3
RPS28
TUT1
CIP2A
PPP1R12A
NDRG2
ADCYAP1
CD5L
KRT33B
SLC7A5
TRPV1
MESD
MAP1LC3B
HOXB1
TH
FST
TSSK1B
TNFAIP6
SYNJ1
PIKFYVE
MACC1
PML
MCM4
DNAJC21
IKBKB
FAT1
HNRNPK
SCN2A
C1S
SLC7A8
GRN
NLRP1
ABCA4
CYSLTR2
NFATC1
TNFSF14
CAVIN4
ADM2

MIR217
NIN
SMAD6
HBA2
DNMT3B
SULT2A1
USP1
RAB18
HEY1
ADAMTS3
CYP17A1
HSPB11
RAN
CYSLTR1
NLRP6
PMEL
CXCR6
CDH3
MIR132
COL22A1
DISP1
NOD1
GK
LRRK2
MBOAT7
VTI1B
AIRE
MT-TS2
MT-TQ
NES
CD38
KIF27
EPRS1
ASB7
CACNA1C
HUWE1
SLCO1B3
PIK3R1
NLRP5
RICTOR
FZD3
COQ5
MIR34B
KRT17
ACTA1
DUSP19
ZDHHC9
DVL2
SLC25A3
RLN2
THOC2
BBC3
IRF8
SLC7A6
XK
BID
LCN1
NDUFB11

CYBB
NEDD4
MLANA
TERF1
PCDH19
BAIAP3
FAM186B
LAP3
AREG
BACH2
MVP
ACACA
KIAA0930
MECOM
LIN28A
NCOR1
SLC25A20
NTRK2
RLN1
CD1C
ADSL
TIMP4
NAGA
ATF4
P4HB
PIAS1
ANGPTL2
HEY2
NFKB2
SYK
ADIPOR2
REG1A
ITK
HOXA7
BLOC1S2
MYO6
HSP90B1
TXNRD3
PRKCE
FRZB
ATP2B3
NR4A1
SPTBN1
PRPF40A
COL18A1
CFAP36
PTPN3
SLC36A1
HARS1
SIL1
CALM1
MCM6
ADH1B
ANXA4
ST2
PEPD
MNX1
UGT1A7

NDRG1
CALM2
H2AX
MIR133A1
CD1A
TBX15
FABP12
XPO1
LAMC1
PPIA
ACAD11
NDUFS8
CGB3
NOG
LRP6
SLCO1B1
ASCC1
CACNA1D
TBXA2R
JUNB
SLC11A2
IFNGR2
NALCN
CALCR
RLBP1
PDCD4
KLK3
SLC26A2
TP53BP1
PINK1
JMJD6
TAPT1
HOTTIP
DDC
CNGB3
PTK2B
NPEPPS
MCAM
TNFRSF11A
PDPK1
SLC45A2
SLC2A4
AFF4
NAIP
SLC1A7
WNT7A
E2F1
PAWR
DPYD
MIR30B
TRAF6
TEFM
CEP170
MEF2C
FHL1
SELENOP
ATR
MIR361

CROCC
POLR1C
MIR205
COL5A2
MIRLET7B
VSIG4
CASP7
RAPSN
RHOD
PFN1
NLRC5
TACR1
LOC108281186
GHRH
TCOF1
INTS1
SNHG6
CCDC120
MIR30D
SCGB3A2
INCENP
PRNP
MYH3
PRKAR2B
CRH
PLCG1
MGAM
M6PR
HNRNPA1
ATP9A
HSD17B1
CSF3R
SEPTIN6
RARRES2
GRB2
ABCF1
SORL1
RCC1L
PLD3
WG
MATN3
ADAMTS1
RXFP2
CYP2J2
IL18R1
STMN1
SMYD3
CCDC82
MIR340
KIF21B
SPTLC1
CHKA
MUS81
HOOK1
VCAN
IRF6
HES1
DYNC1H1

ARRB2
RNASE2
AP1S2
CARD8
PLOD3
MMP10
SFRP2
MIR363
MOCS3
CXCL6
TBXT
SLC30A8
HLA-DRA
CYGB
KCNH1
HPD
DBNL
PKHD1L1
SMAD9
LRBA
CDH8
SERPING1
NTN1
ID2
NME3
FKBP5
SNAP29
ATP2A2
KRBOX4
UBA5
IREB2
SMURF2
MIR103A1
PCCB
F12
CD19
CHST14
CCND2
TNFRSF25
IGFBP7
GHSR
MIR152
MAP3K5
MED23
MIR24-2
MIR199A2
ASPH
IL10RA
LXN
CAV3
EXOSC10
F2RL2
MIR183
ATP5F1E
IRF3
FAM50A
DBN1
KDM6B

GRM1
WDR48
LRP1
ASIC2
MIR33A
MIR16-1
NUBPL
BMI1
SMO
MCM7
FAAP100
ADAMTS4
TRIM33
MIR9-2
RUVBL2
CYP2B6
ARNTL
DAP3
F2RL3
MAD1L1
IRS2
GSTA1
DNAAF1
CBL
SGK3
B3GALT6
CCDC78
TM2D3
LPCAT1
CIITA
MOCS2
PLCD1
SFN
TP73
FECH
SLC16A2
L1CAM
IL13RA1
PCSK2
TPT1
SNRNP70
SAMHD1
GDF1
GALC
DCLRE1B
ADRB3
RHBG
PRKRIP1
RAB3GAP2
CST9
TSHR
P4HA2
ATP1B1
DZANK1
SERPINB3
BHLHE22
LPP
APP

LMAN1
CDK20
PRDX6
PKD3
SPINT1
FAAP24
SOX6
CS
INPP4A
TYRP1
ACAA1
CSNK2A1
EXTL3
SLC2A6
TRA
ENPP3
IBSP
B4GALNT1
MYL2
NAT1
IFNA10
H3-7
PALS1
PRPH2
HLA-E
MTUS1
HBB-LCR
MMP16
FOXG1
SPAG17
RCN2
KIR3DL1
TCN1
CUX1
RP2
HNRNPUL1
SDK2
ICOS
HYAL1
RXFP1
PPP2CA
HNRNPC
MC1R
VAMP8
DHFR
WWP2
COQ8A
INPP5K
TXNIP
CDKAL1
MYBPC3
CKAP5
ERG
ODC1
FZD4
IFNL4
IL21
DHRS11

HDAC3
NPR2
AARS2
FAAP20
ACADM
ALDH3A2
HSD17B13
ALPI
EHF
NRIP3
RECK
CLDN5
WWOX
WNT9A
PDIA3
DNM2
SMCHD1
LIF
FABP5
SULT2B1
CENPB
GPR182
SERPINB2
KPNA2
RNPC3
EIF2B4
RORA
KAT8
CYP4V2
HELLS
RASL12
AMMEC
CAPN1
ARRB1
LAMA4
FSHB
XRCC5
PFKP
EIF2B5
AHSP
DECR1
P2RY12
SULF1
PROCR
AQP8
LIN7C
SLC20A2
MLC1
GSTK1
IL34
CXCL14
CPE
B4GALT1
PDE5A
BAZ1A
AFG3L2
UBA52
MIRLET7C

GRHL2
EEF1A1
PYY
RAMP2
CLN3
NPC2
FNDC5
PIGR
MYO1B
RBP1
PLCG2
LMO7
LTB4R2
HADH
CCDC160
H2BC21
ING4
INHBB
RPLP1
SULT1A3
C1QTNF6
FCN1
PTMA
SRP54
TNS3
NPY2R
ITCH
HIPK2
AQP9
TNNI2
MYH1
DIABLO
RRM2
KRT16
TLN1
SUCNR1
ACKR1
IFNAR1
UBR5
RCOR1
SYVN1
ARSH
A2M
MGMT
TCF7
FADS1
LIN28B
CEP295
SPINK5
CD83
IGFBP6
CCL21
DTNB
SDC2
ANXA3
CNTRL
CD248
ITGAE

TARS1
HIVEP3
HOXA11
ROM1
RIPK1
STIM1
PROS1
CSTB
ACTN3
TTLL5
TFF3
DCLRE1A
ATL1
ASIC5
PCM1
FOXA1
TFAM
GNAI2
MYOM2
RYR2
DNAH6
LTBP2
SLC5A5
IL37
SPART
RAPGEF3
MCTP2
RING1
PDE6C
SLC29A1
PCSK1
MIRLET7E
GLP1R
CD1D
TULP4
MIR186
FUT2
H4-16
CTNND1
TENT5A
TIA1
OLFM3
OCA2
CPS1
TARDBP
MAT2A
ACAD9
YWHAZ
FERMT2
BAG1
LINC00667
SSTR2
VAPB
ADH1A
IL21R
RNF220
NKX6-1
LYVE1

TPM1
KDELR2
DCTN1
RELB
RIOK2
UTRN
HIF1A-AS1
OPTN
FRTS1
CD99
IFI27
MIAT
REV1
SLX1B
SLX1A
GIPC1
ANG
MIR96
SLC1A4
SLC13A5
CCT4
PMPCA
IHH
IL2RG
VKORC1
CLOCK
SMAD1
CD47
TYK2
BAG3
S1PR1
TRPC3
TNXA
MAGEA6
MIR486-1
GAD2
EMD
IFNAR2
HSF1
DNAAF9
RASAL1
ANGPTL4
FKBP1A
KCP
MIR197
RDX
MIR382
HTR2A
MALL
CLDN2
SHANK2
AIF1
ZFP36
HSD11B1
CLASP1
AHCY
PPOX
CDCP1

MIR495
ZC3H12A
HSPA1L
SLC6A3
GP2
FDFT1
NEU3
PPP2R2A
PDHX
SBF2
MIR125B1
XPC
CDH17
PHC3
CLUAP1
HCP5
HSP90AB1
PIP4K2C
KLF5
COL6A2
PRKAR2A
RPL24
DOK5
SUV39H1
UNCX
NME2
CETN2
SLC4A8
IMMT
TLR8
MIR125B2
CALB1
MORC2
GPX4
RSPO3
RNF111
HR
ADCY8
GNAI1
ERF
KPNB1
EHD1
EXOC7
CD177
MAGEA1
ABO
TPI1
MIR497
DNM1L
RAB32
ITPR1
ACP1
ACADS
TRPS1
IVD
RPL7A
SNRPA
ADIPOR1

MTDH
ATG7
HEXA
GLB1
SESN2
SGO1
ATP2B1
UBE3C
SEM1
CELF2
MIR26B
MIR506
TXNDC5
CCN5
CAMK1
EPPK1
DIS3
MAP3K1
CPQ
NKX2-2
CYLD
FAF1
ITGB6
BTRC
IGES
FAM161A
PCARE
ADGRG6
ELK1
KDM3A
CENPC
ACTN1
PRDX5
DDX11
YTHDF3
CAPN2
SLC23A2
MIR184
SAR1A
CTTN
NEDD8
HTN3
MIR324
MIR124-1
SLC66A1
GCH1
GUCA1B
PTPN13
TTLL6
GOLPH3
KAT5
CDX2
SLC35A1
FGB
ESRRA
SLAMF7
TGM3
MAS1

AICDA
CASP2
EPPIN
HRH1
FERMT1
UQCC3
TET3
PGRMC1
TCF21
RIT1
VASH1
MCM2
PEBP1
CALCRL
EIF2B2
TTL
TUB
MYO18A
CAPN5
SMN1
MIR10A
SIRT6
CR1L
OMP
CDH13
EPHX2
MAP2K4
MFGE8
SORT1
CHRNB2
FAM20B
PMP22
NEDD1
MIR455
LRRC34
CAND1
EIF6
BTD
PPM1A
MAPT-AS1
CSN1S1
VLDLR
RGS9BP
KIFC3
S100A10
TNS1
DNAH9
DNAJC13
NUP210
ATP6V1E1
LIPF
ABCG1
POC1A
LIMA1
BSCL2
METTL3
ERVW-1
MIR101-2

ADCY1
DHX30
BIRC2
TNFRSF8
MIR499A
SNX10
ACOX1
ICAM2
COX4I1
MAPKAPK2
TOR1A
IPO7
PI4KA
ANK1
TSPAN7
SLC25A12
USP8
GLIS1
TSIX
ADAT3
FKBP10
IMPDH2
ALX3
CCT2
MIR107
RANBP1
MCHR1
SEPSECS
ECT2
GPRC5A
SSPN
FZD7
WNT10A
SGCB
CCL1
PTTG1
FMR1
DRD5
TNFRSF10D
MT-RNR1
BCL10
TUBE1
TREM2
TNFRSF9
GLUL
SEPTIN2
HBS1L
MIR29B2
PCCA
TPM3
FADS2
GARS1
CBX5
AHRR
SOBP
PITPNM1
ITPA
CCL19

CNR2
VAMP3
H6PD
GCKR
ARG2
DNMT3L
LTC4S
DCLK1
SPHK2
MIR301A
AKR1B10
CXCL3
CMBL
ST3GAL4
LSM1
FARSA
TPM2
MSBP1
TMPRSS6
FBP1
GNMT
ALDH6A1
HEPACAM
HSPB2
TRAF3IP2
BLOC1S4
CLIC1
POC5
MESP2
HMCN1
ENTPD2
CARS1
SSB
ANTXR2
FZD6
AEBP1
MSRA
PRDX2
HYOU1
SETX
ASNSD1
MAN1B1
ESYT2
EFNB2
POGLUT1
MIRLET7I
PLXNA1
NUMB
EN1
RPS6KA3
LAMA1
SLC24A5
LFNG
GPR161
DDIT4
CFAP418-AS1
CR2
PTGIR

NCAPD2
CNGA2
PTGES
FLOT1
PIN1
MIPEP
CAPN15
GPNMB
PANK2
IL12RB2
EIF3A
DNAJB12
DKK4
PIP5K1C
PYGB
MT1G
ZBTB16
DLG1
FAR1
ADAMTS5
MIR542
MIR509-3
IL32
EXOC3L2
SULF2
IL31
ESRP2
CBR1
NAGS
ST14
ACADL
STX6
SDC4
RASSF6
TBCE
HSPB7
SLC25A15
S100A11
NIBAN1
GPC1
LUC7L2
MMP11
MCOLN1
ANGPTL3
PSME1
SLC8A3
RUVBL1
HBG2
PRPF31
GAD1
TMEM30B
EIF5A2
MIR103A2
SERPINB5
MIA2
PIFO
COX6B1
CEP350

DISC1
MS4A1
PTGER3
NPR3
RPS6KA1
MIR429
NOX5
MAD2L1
RIF1
DEFA1
KHSRP
POF1B
QDPR
SOCS2
UOX
BAD
C1QTNF3
TBK1
IL23A
ACLY
HES7
SLC39A14
MIR92A1
WDR35-DT
LOC112939934
ERGIC1
XPR1
ISG15
ADD3
HPS1-AS1
LOC100500719
ENSG00000188078
CCR8
CD1E
NDE1
FLVCR1
MIR372
CERT1
PRKCH
NSMF
GNPTAB
AGO2
STEAP3
CABP4
MFAP4
MIR326
MIR137
USP11
BHMT
CYP2A6
SLC20A1
ADRA1B
TSPAN1
CA8
LRPPRC
KRT6B
IL15RA
KCNQ4

IL1R2
COPE
PLN
FUCA1
DNM1
HTR6
RFX2
DAND5
EML6
KIAA1755
SNORD118
LOC101928861
AFAP1-AS1
THADA
BPTF
TRAPPC11
BST2
FUT3
WNT2
CEP250
HOXA2
NREP
HEXB
HK2
RMRP
GFI1B
COMMD1
LIPE
DKK2
SETDB1
MUC13
ITIH4
SVIL
SPTLC2
TNFRSF10C
ATP5PO
ANKRD13A
RD3
CALU
DEPDC5
NKX2-4
ADH4
CD33
DEPTOR
CXCL17
CCKBR
TMEM30A
BANK1
SERPINA12
ROR1
COPS5
HMGCS2
DLX5
SEMA4C
KIF13A
ZBTB14
QPCT
MSX1

APRG1
FHL2
ADRA1A
SCN8A
ATP8B2
POLR2A
IMMP2L
PTCD3
SLIRP
FUT8
OGN
FGF18
CCL24
INSIG1
LTBP3
ORMDL3
PNLIP
DGKA
MIR30C2
ATP5F1A
CISD1
GBA2
CUL4B
UQCRC1
PREP
KIF1C
CENPA
DYNLT1
INTS4
CDC37
ALOX12
AP3M1
ATP2B2
MYBBP1A
ABCA5
CD226
MIR299
TRMT5
GATA6
ART1
BEST1
SEMG1
CHST15
PPIB
PRKAR1B
PPARD
UBE2D2
APLNR
TFPI2
CLTCL1
GLIS3-AS1
MIR942
GP5
CDC27
GIGYF2
RPN2
EFS
PDE6A

TMEM70
TET1
RPS13
AGPAT2
OTULIN
DMGDH
GADD45A
IL17RC
PAK1
ZMPSTE24
RAB38
ADGRE5
AZGP1
CAP1
HTR2B
CISH
S100A7
NPY4R
LOC117307477
PSG2
NUCB2
ATAD3A
GALNT17
IRF7
ITPR2
CTSZ
HPN
MYH2
MYH7B
GGA3
NRG1
MFAP5
DNAJA1
DSG1
FOXA2
DLD
FXYD5
TMEM60
PDHB
B4GALT7
MIR608
COL12A1
MIR218-1
DLL3
KIFAP3
ARL2
IGF2-AS
CETN3
ODF2
CETN1
IL27
ITLN1
THRB
AGFG1
TMEM219
BACH1
FOXL1
PLEKHA6

MGAT5
ALDH1A1
PTPA
ARL1
BRSK2
GLT1D1
CYP26A1
TP73-AS1
SYNE1
DYNC1I1
PCAT1
PROC
BTN2A2
S1PR2
CALD1
PDE3A
CEACAM1
SLC22A3
UBR3
SERPINA6
RSPO1
PON3
KIF5B
PTGES3
PDCD5
HAS1
MIR216A
F7
ACO1
DLGAP4-AS1
DDOST
XPO5
GSTO2
SETD7
GABRA1
ANKRD27
CHRNA2
IGHV4-38-2
UPK3B
PRKAB1
SRRM1
COPG1
ACYP1
RPS6KA6
NOP53
MEP1A
FKBP8
ARF4
DUSP1
TFG
FOSL1
H2AC20
SLC35C1
RPL7
MCFD2
DHX35
EGFR-AS1
ADAM8

KAT2B
PRDM1
LRP2BP
SUCLG2
CELSR1
ACVR2B
PSMD10
HTRA1
KRT1
MIR384
ATP5F1B
PDLIM1
SCAI
MARCKS
PDE11A
NCEH1
CACNA2D4
GRK1
RCC1
MAPRE3
NINL
MTMR7
TUBD1
LRRCC1
CEP128
CEP162
SNTN
CCDC18
ATPSCKMT
MC4R
SNHG5
MTO1
WDR61
GAMT
PRDM2
HNRNPA2B1
UBE2N
LMTK2
DNAL1
SNHG12
RTN4
DRD4
FBRS
ALMS1P1
CASP4
NOL3
TBX20
FEN1
MIR484
UTS2R
TDP2
STOM
RARG
SET
SIRT2
AKR7A2
PPP2R1B
PSMD13

IQGAP1
SHISA2
COG8
CNDP2
RAB37
CREB3L1
SNAP25
SLC6A15
AZU1
POMGNT2
CD109
ZIC1
SLC7A10
HSPA4L
SNAPIN
AGA
MXI1
PAG1
PF4V1
CFAP410
HTR1B
ALAS2
ECEL1
NME4
SLC9A2
UGDH
VWA1
IPO9
FCAR
NELFCD
DUS2
SH2D4B
TMEM255A
EFCC1
SLC6A6
PGAM1
PRPF8
TARS2
AHNAK
LILRB4
RHOC
SEMA7A
CGAS
VAV3
FLOT2
SRRT
MIR1246
TMEM100
KCTD13
MCIDAS
TRPA1
LNCRNA-ATB
SSR1
ATG16L1
TMEM165
ERCC5
MT1A
MEOX2

HTN1
RARRES1
ESYT1
BRD3
NCOA7
FOXP2
CRIM1
ASGR2
UGT1A6
SCP2
BMERB1
CSE1L
GALNT11
TBL1X
TAOK1
ST6GALNAC3
PUS1
AKAP12
PIF1
PROK1
TSG101
CTSV
SLC15A4
ITGB8
CPNE1
MYO15A
CDH9
CENPS
TAT
YWHAG
NDUFA9
GSS
LONP1
LGR4
CCZ1
CCZ1B
MIR219A1
PCMT1
COPB1
NR4A3
CCAR2
ABCC5
MIR375
CORO2B
CHD8
ZFYVE9
MXRA5
SRA1
OAS1
AGTRAP
KCNV2
DRC7
RPL38
MYH13
RHOB
BCL11A
MPZ
LTB4R

GPR68
BAG2
RIOX2
TNFSF13
FPR1
DSG2
COL6A1
PIK3R4
PFKL
DYSF
MDGA2
HDHD5
XPOT
RGN
MMP24
UBTF
RBBP4
PRPF6
MIR1908
GBA3
NFATC3
SLC31A1
MAPK11
IL5RA
MLPH
SERPINA4
SATB1
FABP3
MDC1
TMEM80
EIF3K
QARS1
IRS4
FGL2
JUND
SCD5
RAB14
CGB5
IGHMBP2
HGFAC
MDN1
NEK3
EPB42
LAMTOR2
MON1A
FOXL2
NCF2
DYNC1LI2
DSC1
GSTO1
ZNF141
ADRA2B
ADAM12
CYP8B1
MIR362
GIHCG
ATP2A1
CALM3

PRSS23
WNT10B
ARAP1
EIF2B1
MICOS13
NCBP1
ANGPTL8
CXXC5
PSAT1
ZNF674
LTA4H
FCGRT
SLC33A1
POLD3
UBR4
PLLP
DPM3
BNIP2
CTNNA3
SNHG1
LRG1
SNAP23
HLF
PDXK
CAPZB
KPRP
ARC
HDAC5
NR2E3
RPL14
PIAS2
TARBP2
COG4
CKMT1A
SORCS1
SPAM1
MIR615
TNFRSF13C
MIR24-1
MT1H
ZC3H12C
TM9SF2
UBE2A
APOA5
SNRPN
AKAP1
SYNE2
SCAMP2
SRD5A2
MUC20
UBA1
PPP2R2B
ABHD5
KCNA2
MIR151A
NAB2
EIF2AK2
PGAM5

MIR425
WNT2B
DARS1
KRT23
ATRN
S100A16
TENT4A
STATH
ATG14
CAST
TAPBP
PAX3
SLC6A4
DARS2
HMOX2
RAD52
PTPRF
SOAT1
SLC25A10
ADGB
AASS
OLR1
MIR194-1
PKLR
HSPA6
HOXA11-AS
MIR346
ZSWIM7
TOR4A
PRICKLE1
LRRFIP2
ATF1
PARD3
RABGGTA
AP3S1
KXD1
BHLHE23
SEC22C
WASHC4
MIR92B
SHKBP1
KLF2
ASPA
NRF1
UBE2E3
ZYG11B
UTP15
RHOT1
TERF2
GAR1
CLEC9A
MEG8
DDN
CHRD
COL8A1
RPL21
HNRNPD
PPP1R15A

AP2M1
MIR409
AMY2A
GZMA
SYT6
ROBO4
SIM1
WDR27
KLHL22
PLCB3
ADPRH
CORO1A
PIK3C3
PTN
APELA
MIRLET7F2
ADAMTSL2
NRP2
S100A6
YWHAH
NEU2
MSMB
MIR194-2
ELP2
SCRIB
RXRB
TUFM
RPS6KA2
MIR1291
PSMD5
TAGLN2
CHEK1
TANC1
TNFRSF4
CTPS1
MIR493
CTDSPL
TLR6
ALOX5AP
ATP6V1C1
KIR2DS4
RGS5
KRTAP17-1
IL20
TTPA
KCNH2
PHPT1
CDH4
MIR452
ABCF3
MIR7-3
GJA8
LDB2
UCKL1
ZNF526
TPTE2
PTK7
TMEM43

LGALS7
RNF216
EXOSC3
COL6A3
MYO9A
PHF6
SLC9A9
MIR381
SLC30A10
UBE3B
AGMAT
SNHG18
TMEM164
FZD1
TMPRSS11E
CLPS
GATD3
PRPSAP1
NUDT21
MIR744
CCM2
EYS
CHAC1
COL20A1
RPL10A
SMC4
RPL3
RPS15
RPL28
RPL36
MIR302A
SPTA1
B3GNT2
BMPR1B
CYP4F8
SLC9A6
MIR135A2
CRABP2
CPEB4
UBE2B
CARD10
DDX5
PLCB1
CSMD1
SEMA3C
DTL
CLEC12A
SP3
P2RX4
MIR133A2
TPX2
SPRR1B
ADAM9
CYBRD1
ACER3
ZNF23
OPRK1
E2F2

TUBA1B
ALDH5A1
TNFSF4
SLC16A4
HSPBP1
FXN
MIR198
HTRA2
EIF3I
PAX5
MCRS1
S100A2
ADH1C
MAP3K14
ARL17A
PRDM11
MMP17
SPG7
GPLD1
CCDC39
CCDC40
PHKA1
ERFE
HMGA1
PSME3
DGKB
VEGFB
FUS
DNM3
RGS2
SLC25A4
WIF1
CATSPER1
STAMBP
CASP6
ACP3
NOL10
TMEM115
SBF1
PPT1
MANF
IFITM2
C5AR2
PDCD6
SNHG15
RARS2
TGFB1I1
SMOC2
CCDC86
ABCD4
NR2C2
GPR39
MIR193B
LCLAT1
MAGEA9
EEA1
CLMP
MARK2

MIR18B
AIMP1
SLC38A2
MT-TI
MYCL
PTPRU
MIR202
KCND1
FBXO32
RHD
MMADHC
G3BP1
MIR196B
MIR218-2
NPSR1-AS1
CHIA
ARPC2
SEPTIN9
UROS
NSUN5
FBLN2
MTX1
HMGN1
KRT74
ALG6
SMURF1
UGGT1
AP4E1
RORC
TPSAB1
RPL12
BRD9
KLF14
MUSK
ST3GAL6
RAP1A
RPL10
IPO4
SAAL1
CKMT1B
FOXK1
SNORD96B
VIPR1
BRWD1
MIR660
SCX
HLA-DRB4
STBD1
SRD5A3
PGA3
QKI
ACAT1
SEC16A
RNF8
RNH1
SFXN4
SEMA6A
HSD17B12

FAF2
EIF3M
KRT72
MPRIP
TBC1D1
PRDX3
CD207
OIP5-AS1
NFATC2
SPNS2
PKP2
SCN1A
LRRC37A
TMEM87A
SEMA5A
UBB
MSMO1
PTP4A1
DNAAF11
GDI2
RPS18
CCT5
EIF5A
NAP1L1
IVL
SHARPIN
CTSH
PLD2
TRAF3
SLC47A2
BMP10
ADRA2A
RAD23B
TRIM24
KLRD1
PINX1
UBE2I
TBX21
PTPN14
FABP6
PPA1
UGT1A
COX4I2
P4HA1
MYOF
STX5
CCL15
AFAP1
SLC29A2
CD160
ISCU
VAMP2
CAPZA1
DPAGT1
POMGNT1
CLEC4E
PDXDC1
BLZF1

IGKC
CREM
ALDH1A2
HTR3A
HLA-DMA
CTSS
GRK5
PMM1
VAPA
HRNR
ARPC5L
NRTN
PTBP1
PPP2CB
KLHDC2
ALPK2
BICRA
EEF1A2
LOC102724058
ACP2
MYO1A
KDELR1
FLG2
EEF2
NDUFAB1
MYL12A
CPT1B
COPG2
TRADD
FAM219B
TTC36
CLDN14-AS1
ANKAR
HLA-DMB
KLHL42
DSC2
PMCH
MT1F
ETHE1
SYNGAP1
PIK3CB
CKB
RPS14
SRP72
ADGRV1
IPO5
MALSU1
TNP1
COL5A3
RSPH4A
MACROD2
RBL2
CCT3
DLAT
MANBA
RAP2A
NHS
VDAC2

PCBP2
ACTR1A
MYO1D
DSTN
MMS19
NTPCR
TMEM33
GFPT1
CCL13
RPLP0
ZW10
SURF4
CLEC4A
DGAT2
GNGT1
THBS4
ASAH2
RB1CC1
NISCH
LPAR6
ACVR2A
RPS4X
C1orf220
PRPF3
UBE2D3
ANO6
PDE1A
CD74
LUM
UCN
SERPINB4
WDR43
TRAF2
HAS2-AS1
SEC24B
UIMC1
H2AC13
USP6
KLHL41
FUT11
GNA12
DZIP1
LRRC45
TECR
RPN1
ARCN1
EEF1B2
PDIA6
RPL32
RPL34
EIF3E
PGRMC2
STAU1
PKN1
CUTA
CRAT
NAV1
SUMO2

TES
ATP1A2
NEK7
HLA-S
NPL
SLC2A1-DT
EVI2A
ATP5MF
CEMIP
TRPM5
TUBGCP6
KCNK1
LOC116158507
ERP29
TP53COR1
NNT-AS1
RYR3
BCL7A
HAS3
MAN2A1
HOXA10
SPTBN2
DOLPP1
ASH2L
TAC3
NOP56
MAVS
ODAD1
WNT5B
RAD17
SPAG9
TM9SF3
ADCY9
IRF9
ORM1
AP2B1
RENBP
TRIB3
MMP25
SUMO1
CDIPT
UBE2D1
MGLL
BRD2
CSTA
RPS12
MYL6
PABPC3
CLPTM1
SRP14
ATP5MG
CIDEC
PCDH15
SDHAF1
CLC
DUOX1
CEP78
NRXN1

SCNN1D
SMN2
RPS3
CIC
DIDO1
POM121L12
CFAP46
AHCTF1
ALG14
GOT1
SAMD9
RPL22
TMEM256
MIR450B
P2RY4
DCK
CCL8
COL11A1
DEFA3
VMA21
TNKS1BP1
ADAM28
PIP
STAP1
IL27RA
PDIA4
HNRNPM
STOML2
STRAP
TTC27
RTRAF
TMC6
MYOZ2
CREG1
NUFIP2
RNASEH2A
RALA
GNAQ
NTAN1
CNFN
ARL4D
YARS2
HIGD2B
FER1L4
ZFAS1
SNHG3
METTL7B
AKR1C4
GM2A
DNAJB9
GTF2H2
HUS1
RPS3A
EDIL3
ACTR3B
LOC110806262
NECTIN3
TNR

FDPS
AGBL3
PRM2
RBP3
TOM1
KIR2DL3
SEMA6D
SEC23A
PABPC1
YME1L1
SPATA5
EIF3F
NCAPH
MOV10
ACSL3
RFC5
SRSF9
PABPC4
UPF1
IPO8
MRPL11
TNRC6B
RPL37A
PGAM4
STRBP
ERH
PPP6R3
PCBP3
EBNA1BP2
MRPL41
ARL6IP4
EXOSC6
PRRC2C
H1-10
BCAR3
PLXNB2
HNRNPDL
ATP11C
AKAP6
AMBRA1
CUL1
DPY19L2
ANGEL1
ZFAND2B
C4orf19
KATNBL1
FAM221A
CCDC74B
IFTAP
PHAF1
PSME3IP1
CAMTA1
HGS
ST6GAL1
SP100
GABRB2
ASCL1
CHRNA7

TRAPPC3
GNPAT
POFUT1
ZFHX4-AS1
LINC01111
USP7
MAOB
BCKDHB
PPL
RPS2
TRIM4
ACR
MEIOB
PMAIP1
MIR129-2
SH2D1A
GLCCI1
SERPINE2
ASIC3
ELF5
ANGPT4
HEPH
AP4M1
ABCC11
FSTL3
STK16
ATP2B4
MIR675
MAP2K7
DIO2
EIF3L
IL36G
IL17B
HTT
ALG12
MIR9-3
GGT3P
MIR187
DYNLRB2
ITGA7
FGFBP1
FGL1
TEX14
MS4A2
TNKS2
TACR2
SKP1
DCTN2
COL6A5
KIR3DS1
RAB3B
TK2
ENGASE
IL3RA
CAV2
GTPBP10
GPHA2
SQLE

EN2
CTF1
IL17C
IMPA2
CCT7
HACD3
MCAT
ALOX15
GP6
RGMA
CH25H
LIPK
PNPLA5
LIPJ
LIPM
SLC27A5
PCSK1N
C7
CBLL1
IFIT2
SEC31B
LINC00871
C13orf42
HBQ1
SLTM
TNNT3
GFI1
UBA3
ARF1
NORAD
EREG
BAMBI
HTR1A
IL18BP
CD1B
SRSF1
RAB5B
LMAN2
NAP1L5
MRPS30
SLC35A3
CBX4
RCHY1
SPRTN
CCNG2
SNORD42B
GRIN2B
SH3PXD2A
DUSP8
EHD3
SUGP1
FAM32A
C19orf38
LRIG3
AFDN
CRPPA
CHKB
SLC25A17

TPH1
SEMA4B
HOXA6
PMEPA1
CELSR2
SNU13
PTPN6
TEAD1
P4HTM
BAIAP2L1
ENTPD4
SNRNP35
GYG2
NOL11
DNAAF5
NPLOC4
GRHL1
SS18
GGCT
MIR124-3
MYO1C
KLHL10
TEX11
USP26
TEKT1
AKAP4
SPATA16
TAF7L
TNP2
HAUS7
VSTM2B
EFCAB9
SCARA5
CAPZA2
SERP1
ID3
SRPX2
RNF4
DCBLD2
DLG4
LGALS4
MIR135B
DLGAP5
MIR485
CYB5A
MIR155HG
CACNA1H
SELPLG
TRIM73
CTNND2
SMTN
TRIP12
SARAF
THEM6
USP2
GDI1
RAB5C
CCNO

NIPSNAP2
CNGB1
MIR454
DNAJA3
ADORA3
THBS3
ZEB2-AS1
DAXX
FAM111A
CDCP2
IMPG2
UBXN11
TAS2R4
H3C1
MMRN1
TRAF1
DUX4
ALDH3A1
SYNM
MMP20
GLRX5
SLC25A38
THSD7A
CYP2A13
VASP
WWP1
AMBN
PANK1
ADAMTS7
SPON1
ADAMTS20
FERMT3
ENSG00000266919
IGSF3
CKAP4
MTREX
CCL25
PGPEP1
ARHGAP35
CCNG1
AK1
CCN6
OSMR
ACTR3
ARPC4
TBC1D15
LANCL1
SVIP
RDH11
FAU
ADH5
SUGCT
NAXE
KIFC1
CFAP298
SEPTIN11
SLC52A3
PCDH11X

GAB2
USO1
VTRNA2-1
POLE2
KATNB1
LTN1
TBCD
RYK
PTGFR
NRON
FGF3
CSF2RB
SRD5A1
CCR9
ANP32B
CRHR2
CNTNAP3B
SNRNP40
MIR128-2
NPTX2
SIGLEC7
BHLHA15
CNOT4
PPP1R35
MAP2K6
LIME1
TDRD15
ELF3
MIR433
MIR574
RANBP2
ARPC1B
CSNK2B
PDIA3P1
FBXO2
MIR627
DEGS1
CARD9
NEFH
DENND1B
AGPS
PIPOX
ALG11
GTF2H1
CALML3
IQGAP3
LDHAL6A
PPP2R2D
SELENON
KLF10
ARFGAP1
ARFGAP3
MELTF
C19orf71
HNRNPAB
ELOVL1
WAPL
STAG2

INPP5D
RND3
LARP1
P2RX1
PRPF4B
OGT
SKA2
KIF2A
TRPC4
PHKG1
ACKR2
ABCB10
ATP5PD
CDK13
PSMB7
CKM
B3GALT4
FCER2
FMOD
COIL
FGF4
SOX2-OT
MTM1
GRM5
EGFL7
ALG3
TRIP4
ATXN2
PRICKLE2
CRLS1
SYTL4
TMBIM6
PCOLCE
GALNT10
EXTL2
HSPA2
STK11IP
SIGLEC8
SLC39A1
USP44
ILF3
RCL1
MTA1
CTBP2
SLC25A24
GMPPA
HLA-F
CNN2
APMAP
SERPINB12
C1orf68
SPON2
CLEC3B
SLC27A2
MFNG
LARGE2
PTPRN
CRELD1

PADI4
SHOX2
ASAP1
FAM126A
KRT15
UNC80
RNASEH2C
GCA
GTPBP3
SERAC1
FAM98C
TMEM256-PLSCR3
MAPT-IT1
MIR4315-1
WASL
BRD1
PXK
SELENOO
TASL
VTA1
RIOK1
BMX
NR5A2
CLRN1
FOLR1
PHB2
AP1G1
AIMP2
TNFAIP8L1
OBSCN
PPP3R1
PRSS3
TIGAR
GPR143
MKRN3
IQCE
DLX6
ATP1B4
EGR2
TNNT1
NUDT1
NBR1
MYBPC1
ZNF384
EXOC3
MOB2
ZBED4
ZNF574
PRPF38B
HNRNPF
SNX9
FAM83D
C1QTNF5
NEK11
MIR136
LINC01138
DGAT1
SDK1

MAML2
CLDN6
POLA1
MIR134
DUSP26
NOP58
MAST2
CLINT1
EMC2
NID2
TNIP1
CASQ2
EDARADD
HAPLN2
SHE
RALYL
PLIN3
TSPY1
CELSR3
TRIM50
PALLD
PSMC1
PSMD6
RNF213
KAT2A
CNP
CHPT1
SH3BGRL2
TMPRSS11D
CYP51A1
NXF1
PDE6B
IL36RN
IFIT3
KLF9
MIR543
EBI3
TSEN34
ZWINT
GCSAML
ZNF761
IGHA1
KLF1
SSH3
NOTUM
ZFAND6
EDAR
MAPK12
ESCO1
SMPD4
LRPAP1
MYO18B
WNT16
EXT1
PLP2
TRPC1
ATG9B
CNTNAP2

MYF5
IL1F10
GSK3A
MIR1228
MARCKSL1
ERBIN
SQOR
RNR1
MYBL2
BBOX1
TNK2
PCBP1
VPS41
FGF14
MC3R
NAA40
CECR7
COL11A2
PTCD1
S100A13
RPS25
AZF1
PABPN1
ARHGAP32
HDGF
SLC19A2
CTTNBP2
HPGDS
SIRPA
XPNPEP1
TRIM26
DNAJC9
ANKH
TSPYL2
GOSR2
SPTB
GNG11
UBE2L3
LOC117600004
ING2
TRIM47
PLA2G1B
EFHC2
RPS16
MAGT1
OTUB1
AKR1C2
MADCAM1
EMG1
SLC25A29
COLCA2
HULC
COLCA1
LOC112449713
PYM1
SEL1L
PDE3B
CLTA

TJP3
MIR153-1
LMOD3
INTS5
MYBPC2
GMFB
GALNT4
CELF4
SNX1
SCG5
USP4
RAB9B
GORASP2
EML4
RNASEH2B
KRIT1
EDA
GET3
DUOX2
NARS1
P2RX2
PRPS2
MTMR6
POLM
MOSPD2
IRAK3
HTR5A
GPR85
MXD3
LMCD1-AS1
HTR1D
PARD6B
ALG13
BNIP3L
MYDGF
ARHGAP9
FLG
MTMR4
PPIC
STK36
KIF23
DLEU2
LOC105375913
BYSL
CPA3
TRPM1
MTAP
DDT
TRAM1
PEG10
CSRP1
RNF11
PSRC1
RAB29
MTFR1
LINC00111
RS1
DMPK

TMEM176A
MPP2
ADNP
TSC22D3
DNASE2
SEC22B
MIR331
LGALS8
CTHRC1
ATP6V1A
NDUFA5
SLC4A9
CMIP
GABARAPL1
KIR2DL1
HNP1
PTPRD
EPB41
TSPO
CFAP57
GYG1
RNPEP
MAP4
RBM4B
E2F3
RGS7
SLC35F5
BRI3BP
RPS27P18
ENSG00000250424
HOXA3
HOXA4
HTR7
IQGAP2
SCRN2
ARIH1
PSMG3
ZNF513
TALDO1P1
GTPBP1
REL
IL22RA1
PHOX2B
SACS
CST6
PPY
KLK2
QSOX1
PRDX4
HOXA5
PRRX1
TRA2B
SRSF3
NDUFA12
ARPC1A
IFI44L
RPS4Y1
MIR491

LINC01436
MIR663AHG
ATOX1
CSPG4
EMILIN1
OS9
PSMB6
ITPKC
EPS15L1
PM20D1
STRIP1
CARMIL1
GPAA1
RSF1
INSL5
ARSL
MIR208A
NAA11
SELENBP1
TPD52
TMSB10
PTPRA
FCGR1A
ANKRD1
P2RY11
TICAM1
CNPY3
TM4SF1
PTGR1
IPO13
SEC61G
HIPK3
NELL2
EXOSC9
POP7
RNF20
KIR2DS5
HLA-DRB5
PLBD2
DEFB127
TXNL1
ADAM22
ATE1
KIR2DS2
LAT
GAS2L2
DNAAF6
RAB19
RNF10
L2HGDH
ODAD4
TMED10
SLC6A1
GALNT2
UBIAD1
SEPHS1
NCOA4
SRSF7

PCDHGC3
MRTFB
BAG4
ATP5PF
HSPB6
SOX5
PIK3R3
ELK3
MIR188
STT3A
FAM3C
GLTP
CAMKK2
USP24
CYP2F1
RGS19
ADAMTS12
SORCS2
NRBF2
FNBP1L
ELK4
LRRC75B
TPI1P1
RPL12P36
PTPRS
TRIB2
ADAM2
ASZ1
CASC15
RTN1
BCAP31
PRKD3
GORASP1
KCNE3
ECH1
TERF2IP
AP4B1
RPS9P3
HCRT
MLF1
ANXA11
WDR45
PEX11A
GOLGA5
GRM3
APOBEC3B
AGR2
USP19
LIPH
CBFA2T2
KRT71
DXO
KRT27
TTC7B
DEFB126
STX2
PTPN2
SERPINB6

PANX1
PTPRZ1
RIN1
HECW1
GRB10
SLC26A7
MFAP2
NDUFA10
ENTPD7
TIMM17B
ICA1
AHCYL1
STT3B
USF1
ZNF787
FHOD1
RXFP4
MAP3K20
DEFA5
CHRM2
TSC22D1
GAL3ST4
PADI2
CPB1
MIR501
NFIX
ELP3
ANK2
MYOM3
SEMA3D
NDFIP2
GAL
HRG
KIR2DL5A
GMPPB
MASTL
LRRC32
KRT75
FIS1
HIP1
MAP1LC3C
SHMT2
ZDHHC5
CAVIN1
NEB
LAIR1
GLRX2
DCTD
HSD17B8
STK3
LGALS2
ACYP2
NETO2
CHI3L2
LRFN1
TIMD4
HEPACAM2
ZNF492

MIR502
SLC39A8
GSPT2
HLA-DOA
MAGEL2
MIR339
MIR95
CLPP
COBL
TFEC
SCAMP1
ODAD3
LAYN
CDR1-AS
FOSB
KREMEN1
DOCK9
VSX1
DPH7
CREG2
PCP2
RNF113B
RPL6
LRP8
BCKDHA
IDE
MLLT3
GPT2
ACOX3
CA7
PPP4R4
RFLNA
MT-TG
PSMF1
ITGB5
SAMM50
WDFY3
GCNT3
WDR83
RGS4
MARS2
DIP2C
FZD9
SDC3
PROKR1
KIR2DS1
NEK4
MIR544A
GCM1
MIR500A
SNTA1
UCMA
C1GALT1
ERO1A
TRIM25
ZNF648
H2AC11
H2AC14

ATP6V0E2
FFAR4
CLASP2
HOOK3
SPATS2L
DEFA6
KIAA0319
HK1
SUMO3
PADI6
PRKAG1
GGH
DSC3
SNRPD3
HLCS
TRDN
SMC6
INTS12
MTURN
EPHB3
PLA2G2D
MID2
NGB
PPM1D
AUTS2
LPGAT1
LPAR2
TOMM40
PHF14
STX17
HMGCL
CCDC103
DNAAF4
CHRNG
SFRP5
FFAR2
MCCC1
GZMM
KIF19
CEP20
RN7SL1
MIR379
P2RX6
ZNRF3
SSC4D
SPDYE9
CDC25C
HAND2
CDK9
WDR5
GABARAPL2
RPL4
OPA3
PAXIP1
KLC1
HSCB
RP1L1
ASXL3

STX10
BCAM
KLRB1
ALS2
NUDC
ASGR1
IDO2
LGR6
OPRD1
ADAM23
ARFGAP2
IL19
CHRDL1
STAG3
ROS1
CSDE1
UFC1
PIAS4
CLCN6
SLC13A4
FFAR3
ADCK2
EIF2A
COL15A1
HBG1
CHD3
SNX14
PRRT2
RXYLT1
SCP2D1
ARAP1-AS2
MUC12
PURA
TAB1
CALN1
TRIM74
STAG3L2
CHCHD4P4
TRIM16
XXYLT1
HOXA-AS2
DNAJB13
ODAD2
PTGDR2
BABAM1
KNDC1
MON1B
KBTBD13
KCNE1
ADRA2C
CLCF1
MIR564
MTHFD1L
SNW1
RBM28
TMTC4
ST3GAL2
DPM2

DOCK4
ANKRD49
RAB11FIP4
URB2
STK32B
CHD6
CDCA7L
ITPK1
NUP188
KCNQ3
CRADD
MATR3
TNNI1
MAGOHB
GOLIM4
MRAP2
SLC2A3
CNTF
ATP6V1F
MRPL12
RPL30
KIR2DL4
MBL1P
DNAJC2
CNTNAP3
POM121C
CNPY1
SPDYE2
SPDYE1
MEAK7
STAG3L1
STAG3L4
STAG3L3
SPDYE7P
SPDYE8
SPDYE14
SPDYE12
SPDYE13
SPDYE15
SPDYE10
WBSCR23
STARD13
PLEKHG4
PPP1R14A
IMMP1L
GUCA1A
MYO3B
CERKL
NXNL1
CCDC96
TFDP3
CCDC172
CFAP97D1
ELMO2
CD7
BAG6
PLP1
NFIC

TMEM176B
FKBP4
VEZT
UBE2V1
USP22
AKAP7
P3H4
RRP9
TNKS
CHRND
TLR10
FGGY
PNO1
WDR45B
ZNHIT1
DPP6
PPP1R9A
EIF2S3
EIF2S2
MYEF2
FAM98A
ODR4
EPSTI1
IK
BMS1
RBM39
LAS1L
TARS3
ITGA11
SLC18B1
TGOLN2
ORM2
IL10RB
SMAD5
TSN
KIR3DL3
ALDH9A1
SPRED2
LRRC8A
CHST2
ECI1
ERMP1
GLIPR2
CD2-LCR
RAMP1
FRG1
SERBP1
CHERP
GREM2
HIBADH
AKAP9
SGCE
CELF1
CPED1
CHMP4B
RAB4A
VPS25
ALAS1

TFDP1
MLYCD
GJB4
PDZD7
AMDHD1
CNTROB
PIR
RAB3IL1
TYSND1
WSCD1
MRPL9
BNIP1
PIANP
PEX5L
YIPF2
PEX11G
SGO2
LINC00261
HLA-DPB2
HNF1A-AS1
SNHG20
CBR3-AS1
ZEB1-AS1
GATA6-AS1
TUSC7
HEIH
CASC11
BANCR
MIR518D
GHET1
AOC4P
GLCE
ACAN
NPSR1
RALBP1
SCG2
GCLM
DPT
CHRM1
P4HA3
TBC1D30
KLB
GDF5
H2AC12
STX7
MIR652
PDC
DNAJC15
LANCL2
GBX1
ASB4
NCF1B
GOLGB1
JRKL
COL16A1
CCT6A
ENHO
SPG11

ERV3-1
RLN3
ENO3
GAS8
CYB5R3
CHL1
MRC2
GPRC5B
VWCE
RNPS1
TNPO1
ST8SIA4
NCL
G3BP2
PNPO
FPGS
CD5
ORC4
MCM3AP
INTS8
KIAA1549
MIR802
CAB39
PSEN2
AGPAT1
TRIM9
UNC93B1
ZDHHC13
HSPA13
SAMD8
MFSD8
CALCOCO1
DYNC1LI1
DMTF1
CTDNEP1
PPP4R2
PAPOLB
TMEM248
METTL16
GARIN1A
NCF1C
GARIN1B
PDK4
EVPL
NBL1
PAOX
VPS13A
IL36A
AAAS
MTERF1
ZNF267
KIR2DL2
SCAP
WDR44
RAB1A
PACSIN3
GRID2IP
RAB9A

ZYX
USP5
TMPRSS15
FCER1A
DPP10
RIC1
MAPRE1
ARTN
SELENOS
PPP1R3A
NRG4
PVR
NKRF
PTPRM
RTCB
FDXR
KRT12
KCNK6
HOXA9
EVX1
ATP5MF-PTCD1
MT-TL2
CPVL
FTCD
NBEA
VARS2
PGLYRP1
COLQ
DNPH1
RBP5
PAGE5
CTSC
GNAL
ACADSB
KHDRBS3
SPATA22
MINDY4
ABCA8
ARHGEF2
PEBP4
PFDN2
RSBN1L
RAP1GDS1
CASP12
SEC31A
FIGNL1
SRPRA
C4BPB
CSRP2
RBM33
SERPINI1
NANS
SLC39A4
TRIM63
PPCS
PFDN4
FGFBP2
SKOR1

NSF
GAP43
GSDME
PHLDA1
ASPN
MBL3P
COASY
GLYAT
GLRX3
NEBL
EXOC6B
MT1DP
PTPRN2
LCP1
SNX2
NACA
KCNE2
HOXC13
PDE4DIP
KMT2E
GALNT12
GALNT1
RBBP5
SLC37A3
VCX
LIG3
PPP1CB
FOLR2
WIPI2
TMEM106B
OAS2
MTPN
PFKFB4
TINAGL1
CMKLR1
RBFOX2
MFN1
KRT4
AK4
PPP1R10
MIR646HG
IHG1
DOCK7
NDFIP1
SCAMP3
TM9SF4
GCN1
YOD1
KDM4B
B3GAT3
REEP5
B4GALNT2
DUSP13
EPDR1
PAM
TRPC5
ACSF3
PCDH12

PKP1
LAPTM4B
TRIM23
KLRC2
MYCT1
PAMR1
VARS1
EFR3A
MAGEC2
COMMD4
TIFA
EFR3B
CDR1
FNDC11
ASNS
SRPK2
GLRX
INMT
PCLO
PATZ1
ORAI2
ZNF277
STEAP1B
CILP
CAVIN2
PRLR
NCF4
TPK1
EXOSC8
DAD1
SNRPD2
DDX39B
COPS6
TNFSF9
TRIM59
CALML5
VSTM2A
ATP5PB
LPAR3
MYF6
NLRX1
IL22RA2
IFNA4
MACO1
RXFP3
FGG
ACTL6B
SMARCD3
ITIH5
BRAT1
DAZ1
SRRM3
IGFL3
SPRR2B
HTR2C
GFM1
FXR1
RPL27A

AP2A1
TRA2A
ACAD10
ILF2
PDPR
UGGT2
EIF3B
RFT1
NEMF
MTCH1
TWF1
CAPRIN1
DDX56
RBFOX3
PTDSS2
TMED2
LRWD1
PTPMT1
YTHDC2
OSTC
PRAF2
TMED9
SCAMP4
SPP2
LSM14A
HACD2
SPNS1
LSG1
LMBRD2
MEPCE
WDR47
GPAT4
PYCR3
WDR91
TCAF1
TMEM258
LRRC47
CAAP1
OBI1
MAIP1
FPR2
GBF1
ABCB5
HERC1
MAPK8IP3
ISG20
PCTP
RASSF2
ABCA6
GUF1
NIPA1
SDF4
VGF
DNAJC10
ATP13A3
ZDHHC14
LAMB4
CD99L2

EFHD2
ZNF746
GPR89A
CAMSAP2
RBMY1A1
TMEM221
FAM241B
H2BC12L
C4BPA
NSFL1C
TRIM13
CLIC4
PTAFR
NPTXR
OLFM1
MIR449A
RAB34
PLA1A
APOL4
BLVRA
TKFC
SYPL1
UBL3
LOC110283621
GPR37
DTNA
RRAS
SLC44A4
UBE2H
TSHB
SRI
BPHL
TFPT
ISLR
SLC26A5
MDH1
GNA13
TIAL1
GSDMD
MTFP1
LOC111365141
P2RX3
CIB1
TTF2
A1BG
S100P
YKT6
SCIN
ECM1
NPC1L1
CRYZ
PNPLA8
RAB2A
PIGS
ITM2C
MFF
GHRHR
COL9A3

CREB5
ACSM5
SUGT1
UBXN6
SLC46A3
TTYH3
PDAP1
CCL3L1
VPS50
CTSF
NEXN
STMN2
CLDN12
STARD3NL
TNFAIP8L2
SLC26A10
PRXL2A
SPR
HINT1
EFNB1
ITM2B
DIAPH3
CYP2S1
COL8A2
COTL1
SEC23IP
OSTF1
MGRN1
NAPA
NAPG
GOLGA7
RAB21
STX12
RAB13
TMF1
MMRN2
JPH4
SLC35F6
RABL3
EMC8
BROX
CYRIB
ETV1
EEF2K
SGCD
ST3GAL1
CYP3A43
HCLS1
MMP21
STK17A
MGST1
KCNK2
SH3KBP1
VPS13B
DBF4
OSBPL3
ZC3HC1
STK31

ANKRD7
CSNK2A2
MALT1
USP15
VAV1
RNASEL
CPD
UGT2B4
ARHGEF4
DUSP5
PDIA2
HPCAL1
PDLIM7
RBPMS
SLC30A5
SLC30A1
CERS2
NPTX1
SPAST
HSPA14
GOSR1
STAM2
TMX1
SCFD1
CLCC1
EPN2
RAB3D
PPP1R12B
PHACTR1
PDLIM3
NECTIN2
ARFIP1
COG3
DHRS7
PHTF2
SLC12A9
GSTCD
NDC1
PCNP
EMC3
SVOPL
CEMIP2
TMEM196
TDGF1P3
MAPRE1P1
KCND3
GPD2
MAPK13
ATP2C1
NFASC
DUSP3
GLP2R
S1PR5
TDO2
HDAC11
ENTPD6
PASK
ITIH1

ACVR1C
PPP1R1A
HS6ST2
RTKN
TPST1
SPOCK1
ANO10
CTDSP1
ABCC10
MKRN1
RAPGEF2
USP18
RAB31
FAM3B
TIMELESS
SNTB2
VPS26A
LAPTM4A
INSM1
IRX1
FASTK
CBLC
PPARGC1B
TOM1L1
PLPP3
ZDHHC20
ZDHHC21
MZF1
RNF149
TMED4
PHLDA3
PAQR3
SNTG1
PYROXD2
ENDOD1
ELFN1
HIPK4
MUC21
COPS4
TMED7
TMEM192
C1QTNF9B
VSIR
ZNF786
UFSP1
C6orf120
GINM1
C4orf3
SMIM1
SELENOF
TRBV7-9
HADHAP1
IFN1
LOC111255642
