# Supplementary material for: Anlotinib Alleviates Renal Fibrosis via Inhibition of the ERK and AKT Signaling Pathways
Source: Oxid Med Cell Longev. 2023 Feb 18;2023:1686804. doi: 10.1155/2023/1686804 (PMC9966823; doi:10.1155/2023/1686804)
Supplement: Supplementary Materials — Additional file 1: the primers used in this study. Additional file 2: molecular targets of anlotinib. Additional file 3: targets associated with renal fibrosis. Supplementary Figure S1: the effect of anlotinib on renal function and fibrosis phenotype in vitro. (A) Dose-dependent cytotoxicity of anlotinib in healthy mice by examining 24-hour urinary albumin excretion and serum creatinine. (B) Quantitative RT-PCR was performed to determine the RNA expression of α-SMA, collagen I in the kidney tissue of UUO mice treated with anlotinib in different dose. (C) Dose-dependent cytotoxicity of anlotinib in HK-2 human renal proximal tubule cells by CCK-8. (D) Human proximal tubular cells pretreated with/without anlotinib for 4 hours were incubated with TGF-β1 for 48 hours. Real-time RT-PCR results showed TGF-β1-induced α-SMA and collagen I mRNA expression in the presence of anlotinib with different dose (n = 3). Results are presented as mean ± SEM. ∗∗P < 0.01, n.s indicates not significant (P > 0.05), n = 3. [file 1686804.f1.zip › Supplymental Figure 1.pdf]

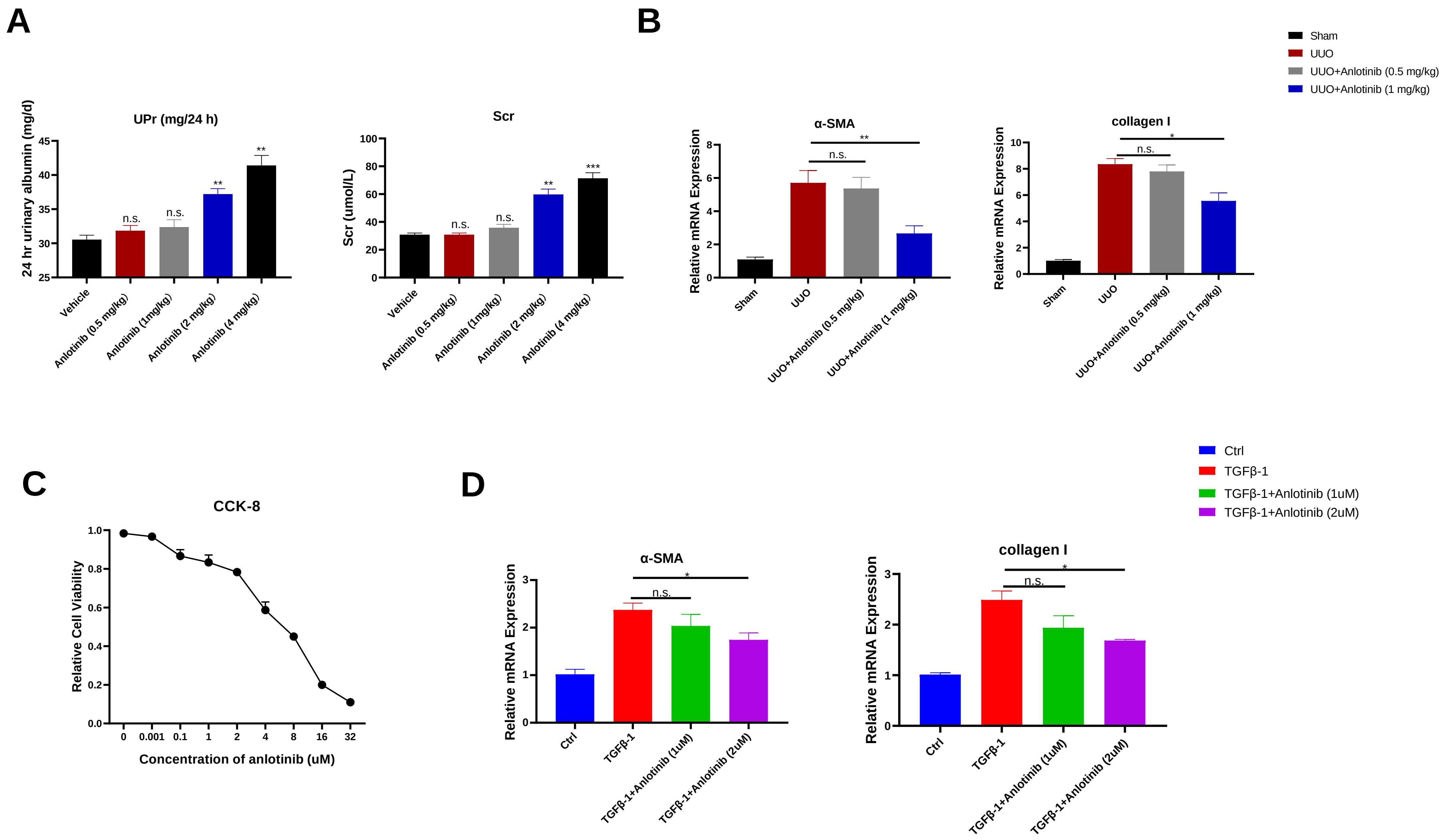
A
UPr (mg/24 h)
24 hr urinary albumin (mg/d)
Vehicle
Anlotinib (0.5 mg/kg)
Anlotinib (1mg/kg)
Anlotinib (2 mg/kg)
Anlotinib (4 mg/kg)
n.s.
**
Scr
Scr (umol/L)
***
B
Sham
UUO
UUO+Anlotinib (0.5 mg/kg)
UUO+Anlotinib (1 mg/kg)
α-SMA
collagen I
Relative mRNA Expression
C
CCK-8
Relative Cell Viability
Concentration of anlotinib (uM)
D
Ctrl
TGFβ-1
TGFβ-1+Anlotinib (1uM)
TGFβ-1+Anlotinib (2uM)
